# Supplementary material for: Representation of features as images with neighborhood dependencies for compatibility with convolutional neural networks
Source: Nat Commun. 2020 Sep 1;11:4391. doi: 10.1038/s41467-020-18197-y (PMC7463019; doi:10.1038/s41467-020-18197-y)
Supplement: Supplementary file 1 — Supplementary Information [file 41467_2020_18197_MOESM1_ESM.pdf]

Supplementary information to

Representation of Features as Images with Neighborhood Dependencies for compatibility with Convolutional Neural Networks

Bazgir et al.

Correspondence: ranadip.pal@ttu.edu

Supplementary Notes

This supplementary document provides some additional analysis results for the application of our Representation of Features as Images with Neighborhood Dependencies (REFINED) approach on the synthetic, NCI60 and GDSC datasets.

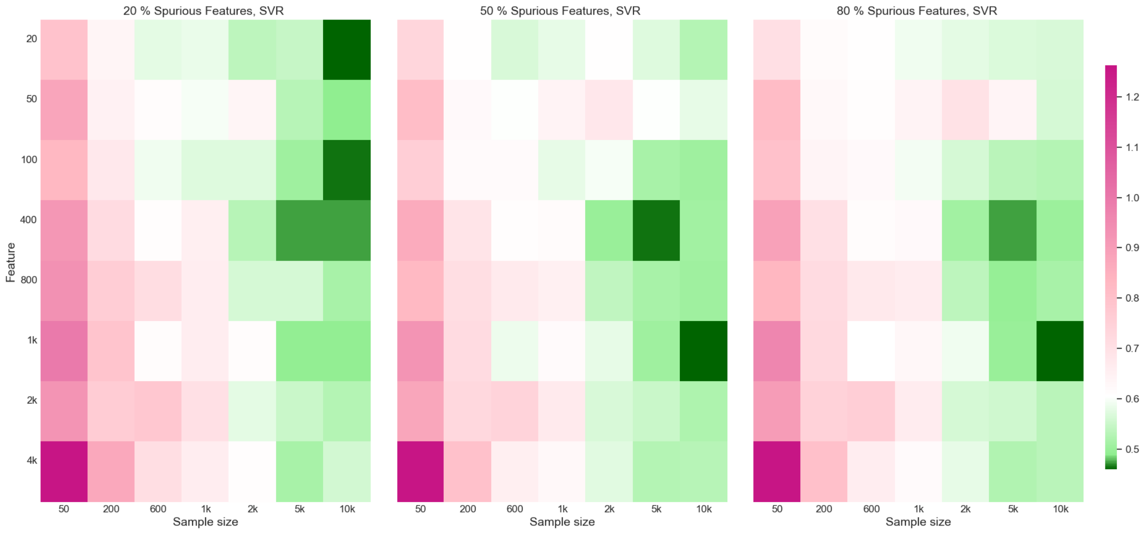

Supplementary Figure 1: Heatmaps representing normalized root mean square errors for support vector regression models built with varying sample sizes and feature sizes containing different percentages of spurious features.

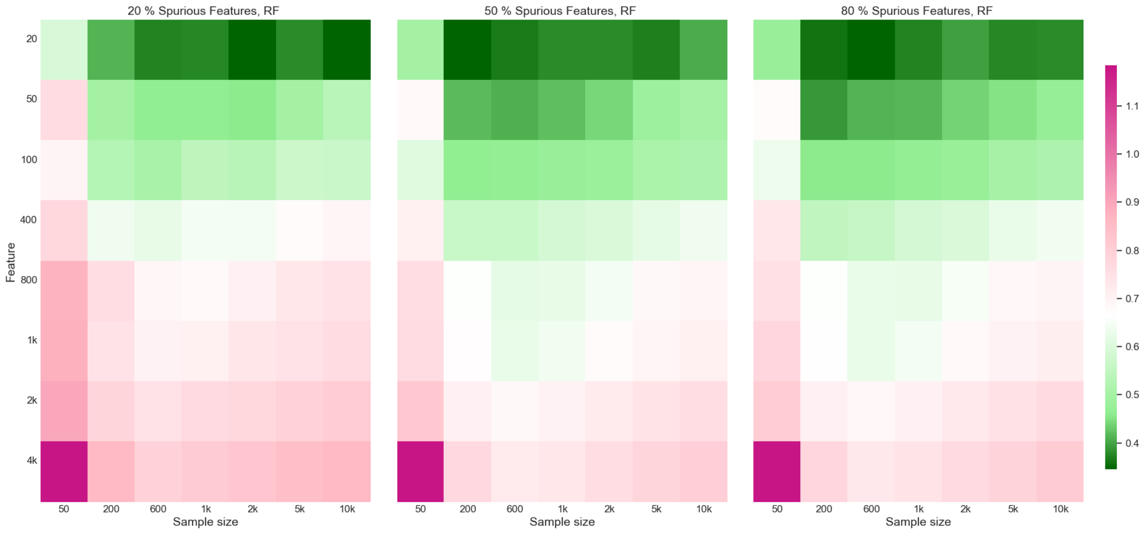

Supplementary Figure 2: Heatmaps representing normalized root mean square errors for random forest models built with varying sample sizes and feature sizes containing different percentages of spurious features.

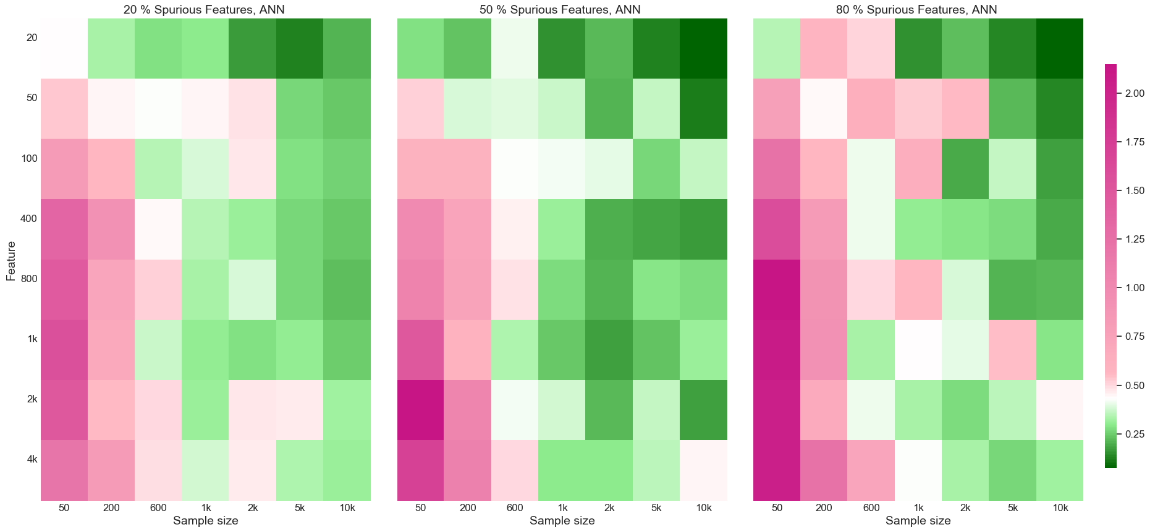

Supplementary Figure 3: Heatmaps representing normalized root mean square errors for artificial neural networks built with varying sample sizes and feature sizes containing different percentages of spurious features.

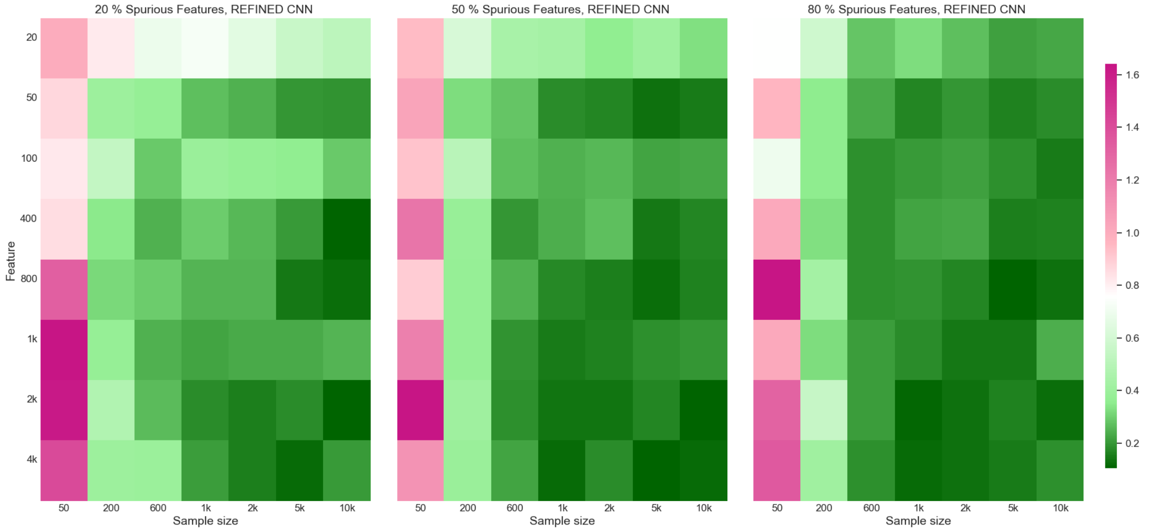

Supplementary Figure 4: Heatmaps representing normalized root mean square errors for the proposed REFINED-CNN models built with varying sample sizes and feature sizes containing different percentages of spurious features.

(a)

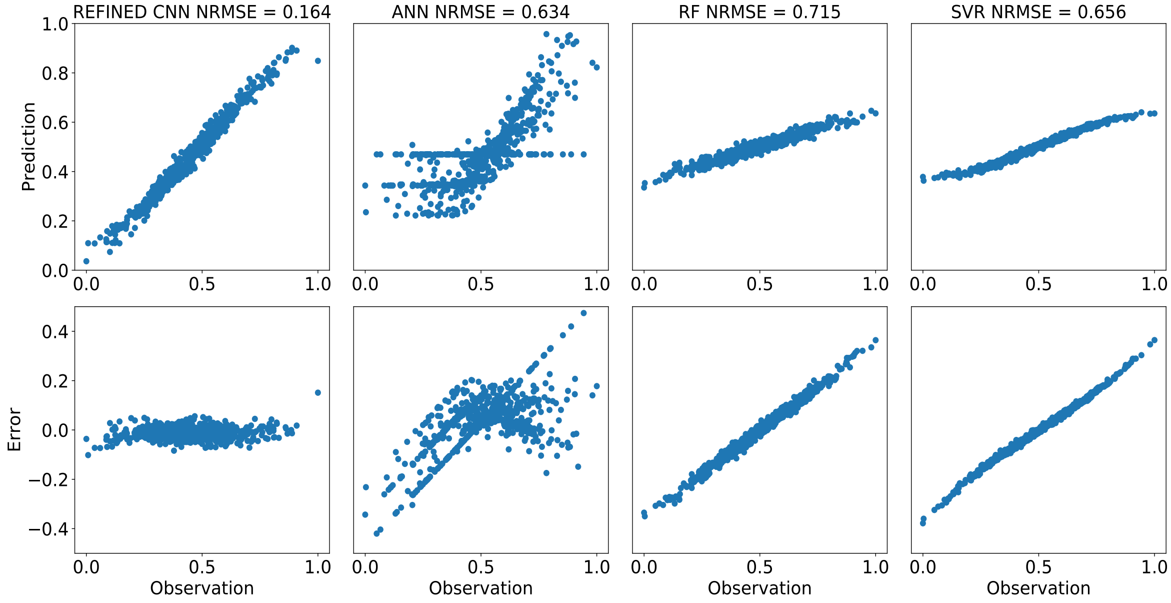

(b)

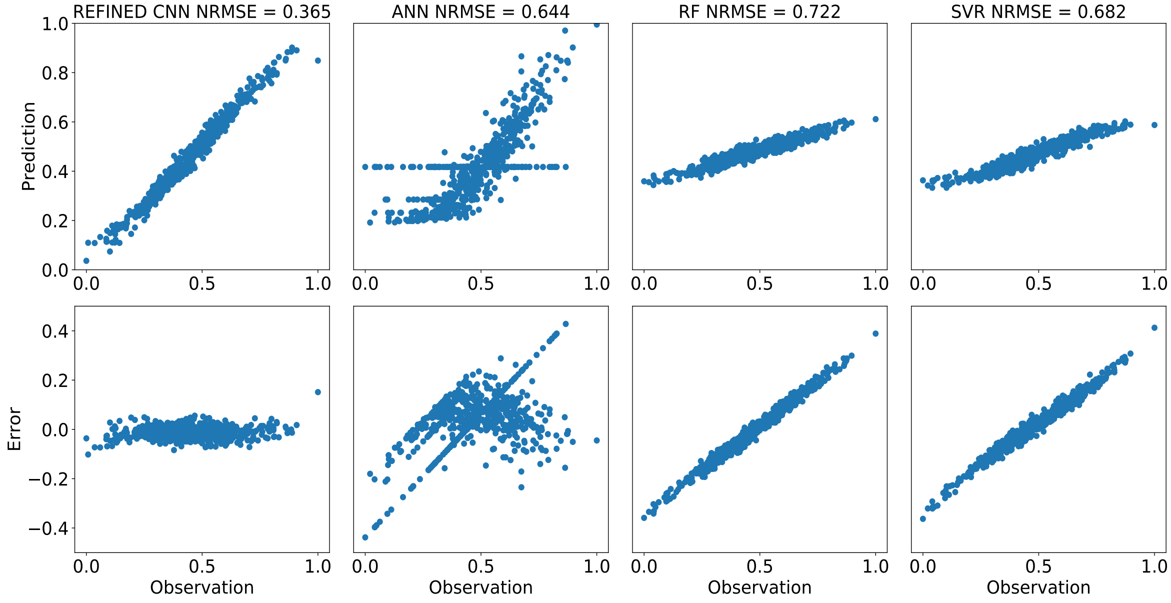

Supplementary Figure 5: Prediction vs. observation and Error vs. observation plots for the synthetic dataset using REFINED-CNN and three competing models with sample size = 600 **and feature size =** 4000 **with different percentage of spurious features.** *a.* Spurious feature percentage = 80%. *b.* Spurious feature percentage = 20%. Note that the scatter plots for REFINED-CNN closely follow a straight line pattern with unity slope indicating the superior predictive accuracy of our approach, while random forest (RF) and support vector regression (SVR) display their well-known tendency to underpredict the higher valued and overpredict the lower valued observations. REFINED-CNN bias is also lower than the bias observed for the artificial neural network scenario

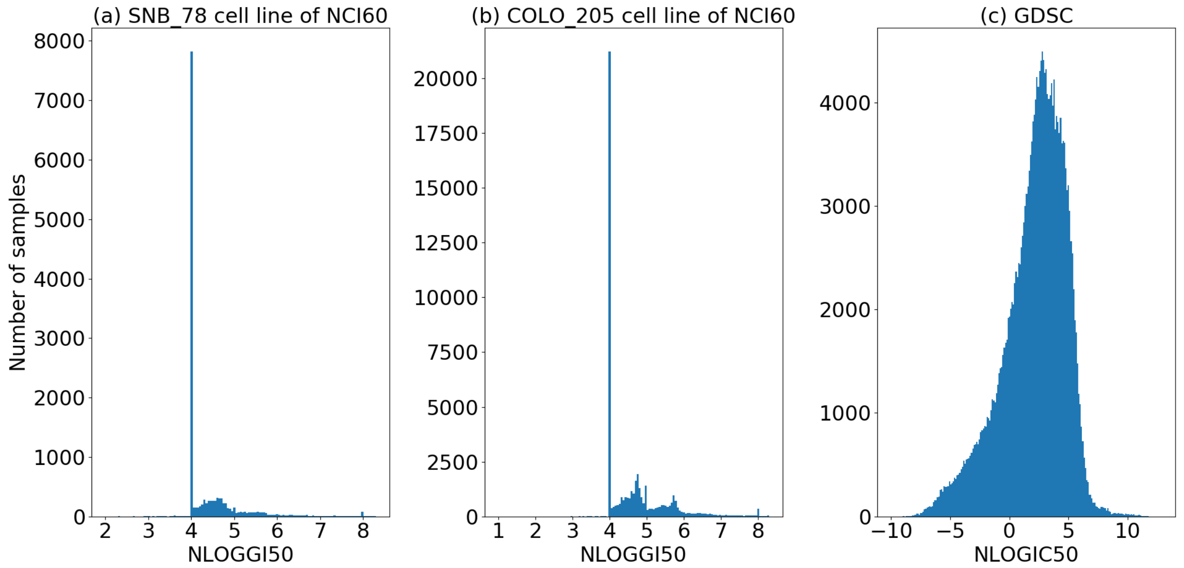

Supplementary Figure 6: Illustration of drug sensitivity distributions for two NCI60 cell lines and for all GDSC cell line-drug combinations. *a* and *b* display the sensitivity distributions of SNB_78 and COLO_205 cell lines in logarithmic scale, where all cell lines with $\geq 10{,}000$ samples (*i.e.*, applied drugs) have similar large peaks at NLOGGI50 ≈ 4 representing the non-sensitive drugs. *c* shows the overall drug sensitivity distribution in logarithmic scale for all samples (*i.e.*, cell line-drug combinations) in GDSC

Supplementary Table 1: Categorization of a drug as sensitive or resistant to the NCI60 cell lines using seven different classifiers *a* three CNN-based classifiers and *b* four non-CNN-based classifiers*

(a)

Cell lines	Random-CNN					REFINED-CNN					PCA-CNN				
	Accuracy	Precision	Recall	F1-score	AUC	Accuracy	Precision	Recall	F1-score	AUC	Accuracy	Precision	Recall	F1-score	AUC
CCRF_CEM	0.725	0.723	0.725	0.723	0.716	**0.752**	**0.751**	**0.752**	**0.75**	**0.743**	0.721	0.720	0.721	0.717	0.708
COLO_205	0.748	0.748	0.748	0.748	0.748	**0.762**	**0.762**	**0.762**	**0.762**	**0.762**	0.734	0.734	0.734	0.734	0.734
DU_145	0.734	0.734	0.734	0.734	0.734	**0.753**	**0.753**	**0.753**	**0.752**	**0.752**	0.713	0.713	0.713	0.713	0.713
EKVX	0.713	0.713	0.713	0.713	0.712	**0.747**	**0.748**	**0.747**	**0.747**	**0.747**	0.709	0.709	0.709	0.708	0.707
HCC_2998	0.710	0.711	0.710	0.709	0.709	**0.758**	**0.758**	**0.758**	**0.758**	**0.758**	0.718	0.718	0.718	0.717	0.717
MDA_MB_435	0.713	0.712	0.713	0.706	0.692	**0.757**	**0.757**	**0.757**	**0.754**	**0.742**	0.692	0.696	0.692	0.693	0.690
SNB_78	0.733	0.723	0.733	0.721	0.672	**0.768**	**0.764**	**0.768**	**0.765**	**0.734**	0.755	0.750	0.755	0.737	0.683
NCI_ADR_RES	0.707	0.712	0.707	0.708	0.708	**0.771**	**0.771**	**0.771**	**0.771**	**0.768**	0.714	0.714	0.714	0.711	0.702
786_0	0.720	0.720	0.720	0.720	0.720	**0.764**	**0.764**	**0.764**	**0.764**	**0.764**	0.713	0.714	0.713	0.713	0.713
A498	0.722	0.723	0.722	0.721	0.722	**0.762**	**0.762**	**0.762**	**0.762**	**0.762**	0.707	0.709	0.707	0.706	0.707
A549_ATCC	0.714	0.717	0.714	0.712	0.710	**0.767**	**0.767**	**0.767**	**0.767**	**0.767**	0.708	0.710	0.708	0.706	0.705
ACHN	0.698	0.701	0.698	0.697	0.698	**0.745**	**0.747**	**0.745**	**0.745**	**0.745**	0.706	0.707	0.706	0.706	0.706
BT_549	0.719	0.718	0.719	0.716	0.711	**0.747**	**0.749**	**0.747**	**0.745**	**0.739**	0.700	0.701	0.700	0.700	0.699
CAKI_1	0.714	0.714	0.714	0.714	0.714	**0.755**	**0.759**	**0.755**	**0.754**	**0.755**	0.716	0.719	0.716	0.715	0.715
DLD_1	0.722	0.717	0.722	0.695	0.642	**0.779**	**0.775**	**0.779**	**0.775**	**0.745**	0.734	0.728	0.734	0.716	0.666
DMS_114	0.681	0.682	0.681	0.674	0.669	**0.736**	**0.737**	**0.736**	**0.733**	**0.727**	0.683	0.688	0.683	0.673	0.668
DMS_273	0.705	0.701	0.705	0.698	0.679	**0.704**	**0.704**	**0.704**	**0.691**	**0.669**	0.762	0.760	0.762	0.760	0.747
Mean	0.716	0.716	0.716	0.712	0.703	**0.754**	**0.755**	**0.754**	**0.753**	**0.746**	0.717	0.717	0.717	0.713	0.705

(b)

Cell lines	LR					RF					SVM					ANN				
	Accuracy	Precision	Recall	F1-score	AUC	Accuracy	Precision	Recall	F1-score	AUC	Accuracy	Precision	Recall	F1-score	AUC	Accuracy	Precision	Recall	F1-score	AUC
CCRF_CEM	0.666	0.672	0.666	0.647	0.638	0.695	0.695	0.695	0.689	0.678	0.685	0.693	0.685	0.67	0.66	0.698	0.697	0.698	0.692	0.682
COLO_205	0.676	0.681	0.676	0.674	0.676	0.697	0.697	0.697	0.697	0.697	0.689	0.69	0.689	0.688	0.689	0.704	0.709	0.704	0.702	0.704
DU_145	0.672	0.681	0.672	0.665	0.667	0.698	0.699	0.698	0.698	0.697	0.687	0.69	0.687	0.685	0.685	0.707	0.709	0.707	0.706	0.708
EKVX	0.655	0.658	0.655	0.655	0.657	0.669	0.669	0.669	0.669	0.669	0.659	0.659	0.659	0.659	0.659	0.672	0.673	0.672	0.671	0.67
HCC_2998	0.662	0.667	0.662	0.661	0.663	0.69	0.69	0.69	0.69	0.69	0.688	0.689	0.688	0.688	0.689	0.705	0.708	0.705	0.704	0.705
MDA_MB_435	0.682	0.687	0.682	0.664	0.65	0.699	0.698	0.699	0.691	0.677	0.694	0.699	0.694	0.678	0.663	0.706	0.705	0.706	0.7	0.687
SNB_78	0.735	0.736	0.735	0.704	0.644	0.755	0.75	0.755	0.74	0.687	0.732	0.748	0.732	0.689	0.627	0.742	0.741	0.742	0.716	0.656
NCI_ADR_RES	0.69	0.696	0.69	0.677	0.668	0.706	0.704	0.706	0.702	0.694	0.7	0.703	0.7	0.69	0.68	0.712	0.711	0.712	0.708	0.699
786_0	0.682	0.687	0.682	0.679	0.681	0.698	0.698	0.698	0.698	0.698	0.695	0.697	0.695	0.694	0.694	0.706	0.706	0.706	0.706	0.706
A498	0.673	0.678	0.673	0.671	0.673	0.701	0.701	0.701	0.701	0.701	0.695	0.697	0.695	0.694	0.695	0.707	0.716	0.707	0.704	0.707
A549_ATCC	0.678	0.68	0.678	0.678	0.679	0.708	0.707	0.708	0.707	0.707	0.699	0.699	0.699	0.699	0.699	0.713	0.713	0.713	0.713	0.712
ACHN	0.66	0.667	0.66	0.657	0.661	0.686	0.687	0.686	0.685	0.686	0.675	0.678	0.675	0.673	0.675	0.683	0.69	0.683	0.68	0.683
BT_549	0.673	0.687	0.673	0.656	0.654	0.702	0.705	0.702	0.697	0.691	0.692	0.705	0.692	0.678	0.674	0.7	0.711	0.7	0.689	0.684
CAKI_1	0.669	0.676	0.669	0.665	0.667	0.691	0.691	0.691	0.691	0.691	0.687	0.689	0.687	0.686	0.686	0.701	0.702	0.701	0.7	0.7
DLD_1	0.713	0.707	0.713	0.682	0.628	0.71	0.698	0.71	0.695	0.647	0.706	0.704	0.706	0.666	0.612	0.719	0.709	0.719	0.702	0.653
DMS_114	0.675	0.674	0.675	0.673	0.669	0.675	0.675	0.675	0.672	0.666	0.671	0.673	0.671	0.663	0.658	0.68	0.682	0.68	0.681	0.68
DMS_273	0.687	0.685	0.687	0.671	0.649	0.712	0.711	0.712	0.701	0.679	0.672	0.681	0.672	0.638	0.619	0.704	0.7	0.704	0.698	0.68
Mean	0.679	0.683	0.679	0.669	0.66	0.7	0.699	0.7	0.695	0.685	0.69	0.694	0.69	0.679	0.668	0.703	0.705	0.703	0.698	0.689

*Bold values indicate the best performances.

(a)

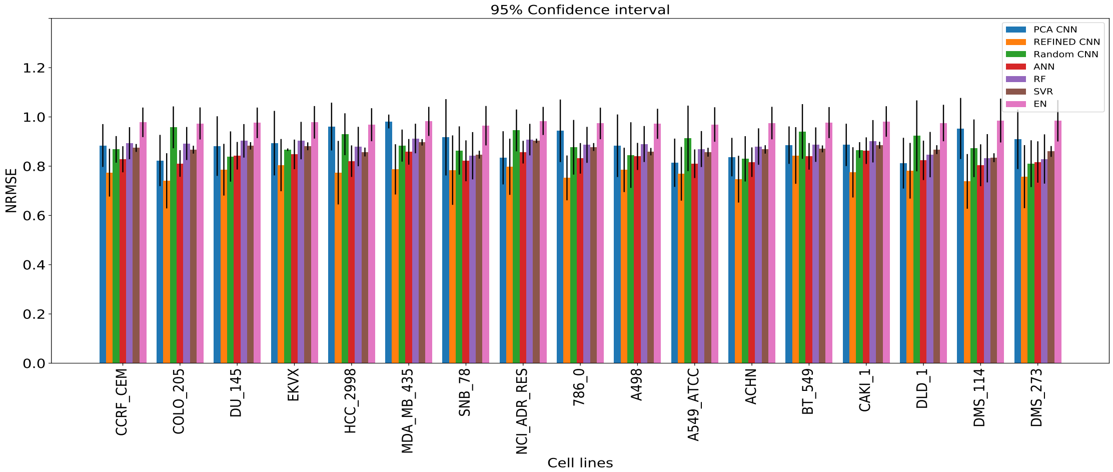

(b)

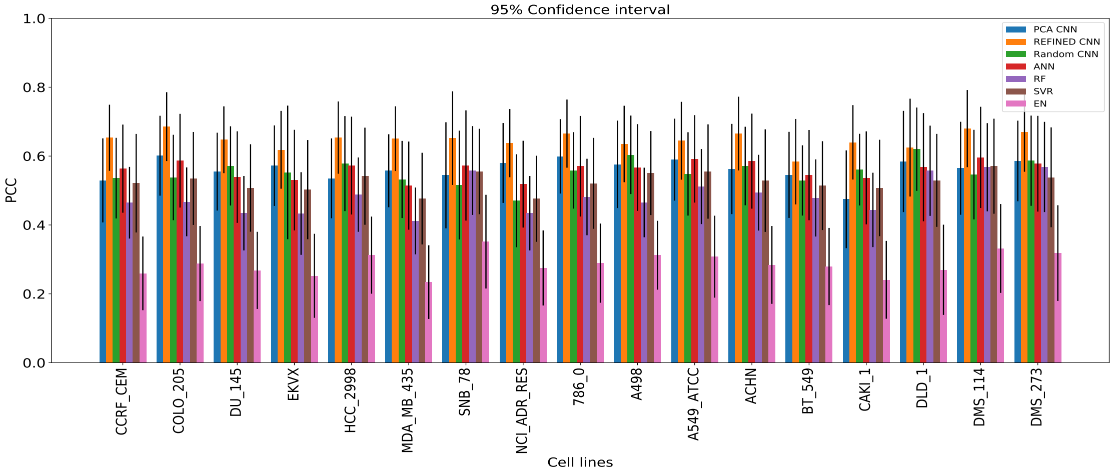

(c)

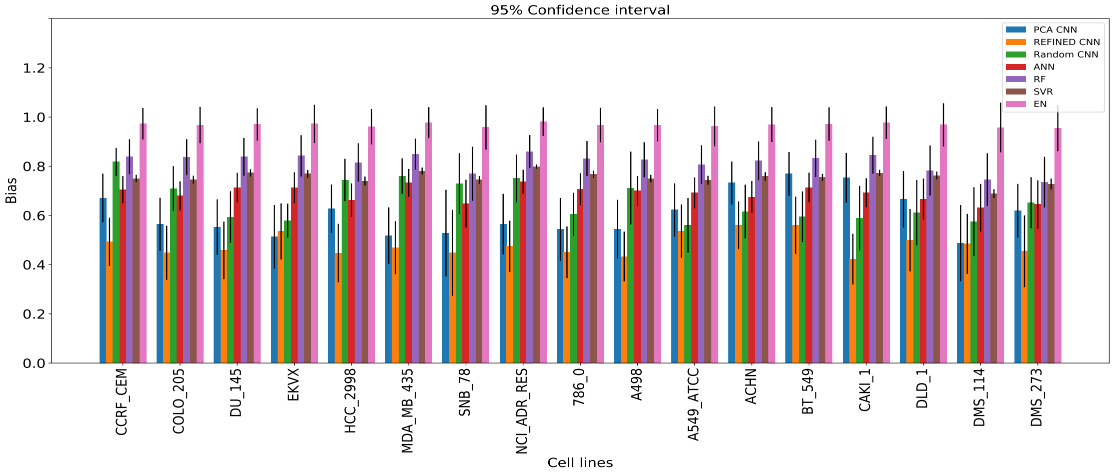

Supplementary Figure 7: 95% **confidence intervals (95% CI) of the evaluation metrics for each model trained on the** 17 **NCI60 cell lines, reported for each cell line per model.** *a.* 95% CI for normalized root mean square error (NRMSE). *b.* 95% CI for Pearson correlation coefficient (PCC). *c.* 95% CI for Bias. The cell line associated with each barchart group is defined under the group PCA-CNN (blue), REFINED-CNN (khaki), Random-CNN (green), ANN (red), RF (purple), SVR (brown) and EN (pink).

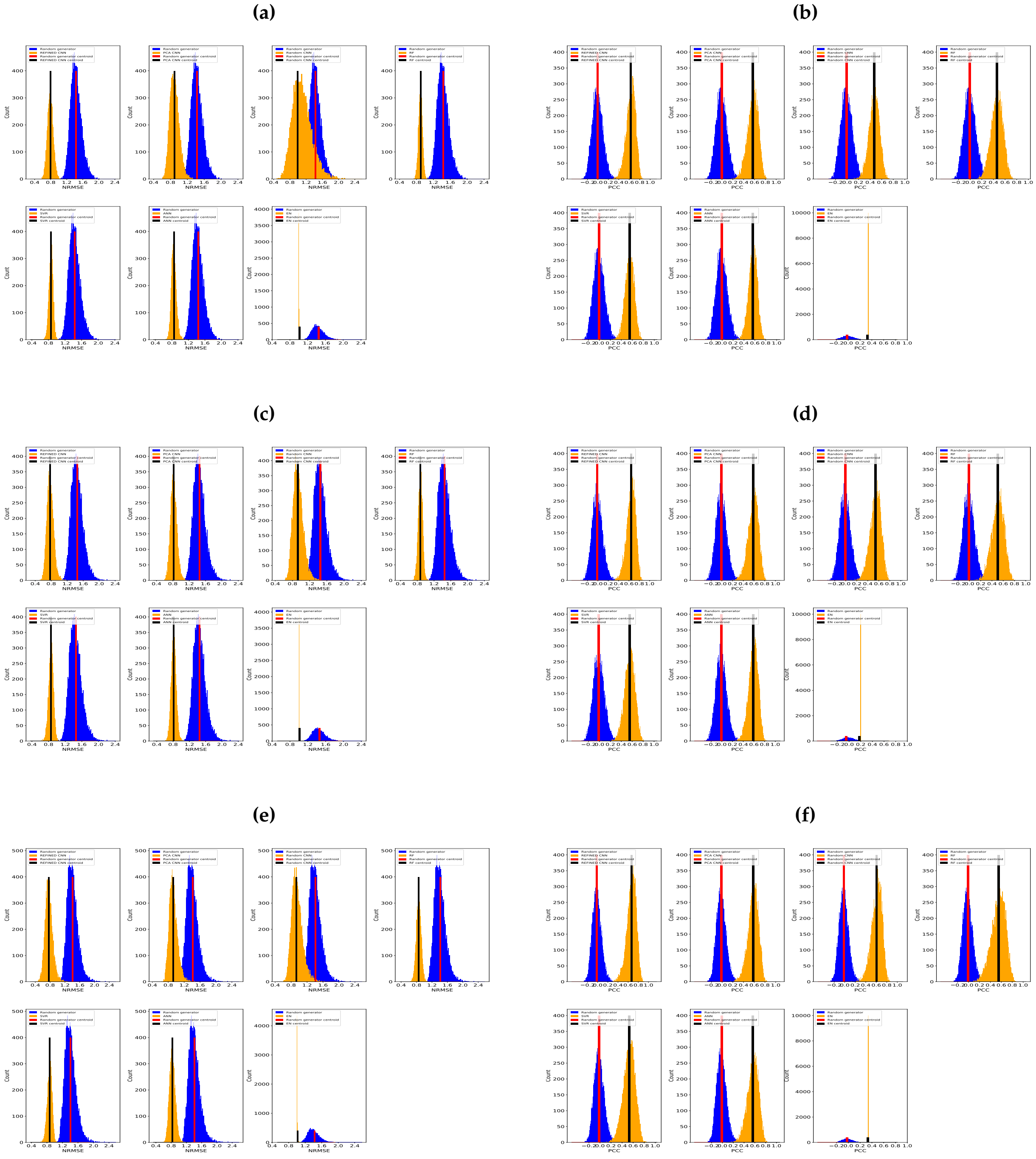

Supplementary Figure 8: Distributions of normalized root mean square error (NRMSE) and Pearson correlation coefficient (PCC) values of all seven models drawn from the Gap statistics test for three NCI60 cell lines. *a* and *b* show the NRMSE and PCC distributions for the cell line A498. *c* and *d* show the NRMSE and PCC distributions for the cell line A549_ATC. *e* and *f* show the NRMSE and PCC distributions for the cell line DMS_273. In each case, the distributions are divided into two clusters and the corresponding cluster centroids are represented as vertical bars.

Supplementary Table 2: Prediction of 95% sensitivity or resistance confidence intervals for seven regression models

Cell lines	Random CNN					REFIND CNN					PCA CNN				
	Accuracy	Precision	Recall	F1-score	AUC	Accuracy	Precision	Recall	F1-score	AUC	Accuracy	Precision	Recall	F1-score	AUC
CCRF_CEM	0.038	0.038	0.038	0.038	0.039	0.037	0.037	0.037	0.037	0.038	0.039	0.039	0.039	0.039	0.039
COLO_205	0.037	0.037	0.037	0.037	0.037	0.037	0.037	0.037	0.037	0.037	0.038	0.038	0.038	0.038	0.038
DU_145	0.038	0.038	0.038	0.038	0.038	0.037	0.037	0.037	0.037	0.037	0.039	0.039	0.039	0.039	0.039
EKVX	0.039	0.039	0.039	0.039	0.039	0.037	0.037	0.037	0.037	0.037	0.039	0.039	0.039	0.039	0.039
HCC_2998	0.039	0.039	0.039	0.039	0.039	0.037	0.037	0.037	0.037	0.037	0.039	0.039	0.039	0.039	0.039
MDA_MB_435	0.039	0.039	0.039	0.039	0.040	0.037	0.037	0.037	0.037	0.038	0.040	0.040	0.040	0.040	0.040
SNB_78	0.038	0.038	0.038	0.039	0.040	0.036	0.036	0.036	0.036	0.038	0.037	0.037	0.037	0.038	0.040
NCI_ADR_RES	0.039	0.039	0.039	0.039	0.039	0.036	0.036	0.036	0.036	0.036	0.039	0.039	0.039	0.039	0.039
786_0	0.039	0.039	0.039	0.039	0.039	0.036	0.036	0.036	0.036	0.036	0.039	0.039	0.039	0.039	0.039
A498	0.039	0.038	0.039	0.039	0.039	0.037	0.037	0.037	0.037	0.037	0.039	0.039	0.039	0.039	0.039
A549_ATCC	0.039	0.039	0.039	0.039	0.039	0.036	0.036	0.036	0.036	0.036	0.039	0.039	0.039	0.039	0.039
ACHN	0.039	0.039	0.039	0.039	0.039	0.037	0.037	0.037	0.037	0.037	0.039	0.039	0.039	0.039	0.039
BT_549	0.039	0.039	0.039	0.039	0.039	0.037	0.037	0.037	0.037	0.038	0.039	0.039	0.039	0.039	0.039
CAKI_1	0.039	0.039	0.039	0.039	0.039	0.037	0.037	0.037	0.037	0.037	0.039	0.039	0.039	0.039	0.039
DLD_1	0.039	0.039	0.039	0.040	0.041	0.036	0.036	0.036	0.036	0.037	0.038	0.038	0.038	0.039	0.041
DMS_114	0.040	0.040	0.040	0.040	0.040	0.038	0.038	0.038	0.038	0.038	0.040	0.040	0.040	0.040	0.040
DMS_273	0.039	0.039	0.039	0.039	0.040	0.039	0.039	0.039	0.040	0.040	0.037	0.037	0.037	0.037	0.037
Mean	0.039	0.039	0.039	0.039	0.039	0.037	0.037	0.037	0.037	0.037	0.039	0.039	0.039	0.039	0.039

Cell lines	LR					RF					SVM					ANN				
	Accuracy	Precision	Recall	F1-score	AUC	Accuracy	Precision	Recall	F1-score	AUC	Accuracy	Precision	Recall	F1-score	AUC	Accuracy	Precision	Recall	F1-score	AUC
CCRF_CEM	0.041	0.040	0.041	0.041	0.041	0.040	0.040	0.040	0.040	0.040	0.040	0.040	0.040	0.040	0.041	0.039	0.039	0.039	0.040	0.040
COLO_205	0.040	0.040	0.040	0.040	0.040	0.039	0.039	0.039	0.039	0.039	0.040	0.040	0.040	0.040	0.040	0.039	0.039	0.039	0.039	0.039
DU_145	0.040	0.040	0.040	0.041	0.041	0.039	0.039	0.039	0.039	0.039	0.040	0.040	0.040	0.040	0.040	0.039	0.039	0.039	0.039	0.039
EKVX	0.041	0.041	0.041	0.041	0.041	0.040	0.040	0.040	0.040	0.040	0.041	0.041	0.041	0.041	0.041	0.040	0.040	0.040	0.040	0.040
HCC_2998	0.041	0.041	0.041	0.041	0.041	0.040	0.040	0.040	0.040	0.040	0.040	0.040	0.040	0.040	0.040	0.039	0.039	0.039	0.039	0.039
MDA_MB_435	0.040	0.040	0.040	0.041	0.041	0.039	0.039	0.039	0.040	0.040	0.040	0.039	0.040	0.040	0.041	0.039	0.039	0.039	0.039	0.040
SNB_78	0.038	0.038	0.038	0.039	0.041	0.037	0.037	0.037	0.038	0.040	0.038	0.037	0.038	0.040	0.042	0.038	0.038	0.038	0.039	0.041
NCI_ADR_RES	0.040	0.040	0.040	0.040	0.040	0.039	0.039	0.039	0.039	0.040	0.039	0.039	0.039	0.040	0.040	0.039	0.039	0.039	0.039	0.039
786_0	0.040	0.040	0.040	0.040	0.040	0.039	0.039	0.039	0.039	0.039	0.040	0.039	0.040	0.040	0.040	0.039	0.039	0.039	0.039	0.039
A498	0.040	0.040	0.040	0.040	0.040	0.039	0.039	0.039	0.039	0.039	0.040	0.039	0.040	0.040	0.040	0.039	0.039	0.039	0.039	0.039
A549_ATCC	0.040	0.040	0.040	0.040	0.040	0.039	0.039	0.039	0.039	0.039	0.039	0.039	0.039	0.039	0.039	0.039	0.039	0.039	0.039	0.039
ACHN	0.041	0.041	0.041	0.041	0.041	0.040	0.040	0.040	0.040	0.040	0.040	0.040	0.040	0.040	0.040	0.040	0.040	0.040	0.040	0.040
BT_549	0.040	0.040	0.040	0.041	0.041	0.039	0.039	0.039	0.039	0.040	0.040	0.039	0.040	0.040	0.040	0.039	0.039	0.039	0.040	0.040
CAKI_1	0.040	0.040	0.040	0.041	0.041	0.040	0.040	0.040	0.040	0.040	0.040	0.040	0.040	0.040	0.040	0.039	0.039	0.039	0.039	0.039
DLD_1	0.039	0.039	0.039	0.040	0.042	0.039	0.039	0.039	0.040	0.041	0.039	0.039	0.039	0.041	0.042	0.039	0.039	0.039	0.039	0.041
DMS_114	0.040	0.040	0.040	0.040	0.040	0.040	0.040	0.040	0.040	0.041	0.040	0.040	0.040	0.041	0.041	0.040	0.040	0.040	0.040	0.040
DMS_273	0.040	0.040	0.040	0.040	0.041	0.039	0.039	0.039	0.039	0.040	0.040	0.040	0.040	0.041	0.042	0.039	0.039	0.039	0.039	0.040
Mean	0.040	0.040	0.040	0.040	0.041	0.039	0.039	0.039	0.040	0.040	0.040	0.040	0.040	0.040	0.040	0.039	0.039	0.039	0.039	0.040

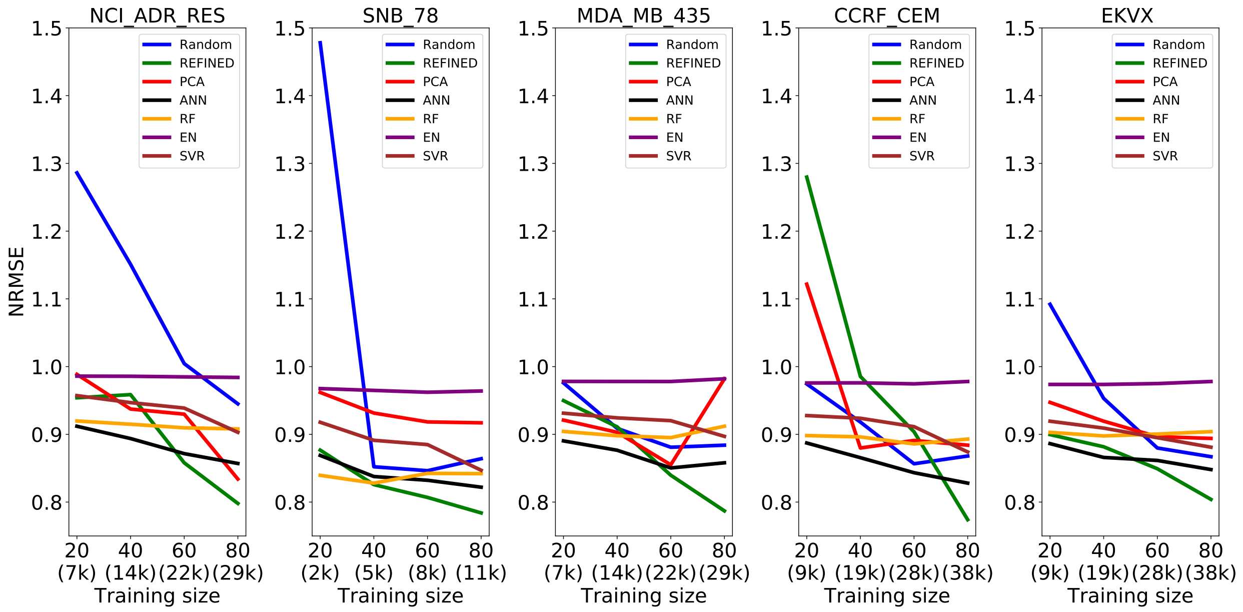

Supplementary Figure 9: Comparison of six competing models with REFINED-CNN for different training sizes using five randomly selected NCI60 cell lines. The x-axis represents the data percentage used for training along with the actual sample size in parentheses. We used the following shorthand legends: REFINED-CNN = REFINED, Random-CNN = Random, and PCA-CNN = CNN

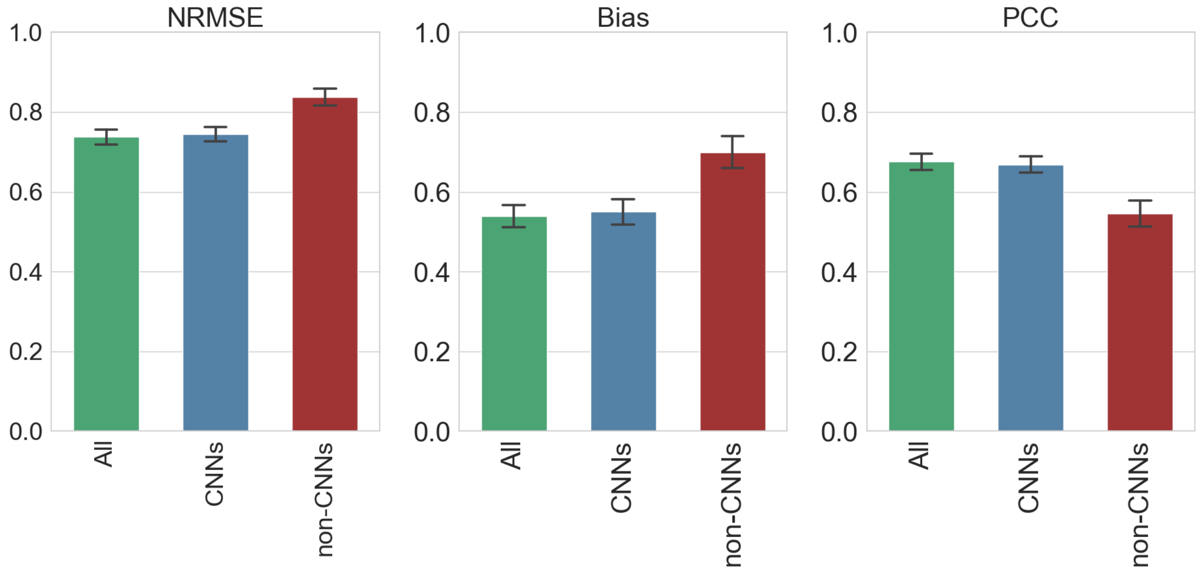

Supplementary Figure 10: Comparison of stacking performance of all predictive models (All in green), all CNN-based models (CNNs in blue), and all non-CNN models (non-CNNs in red).

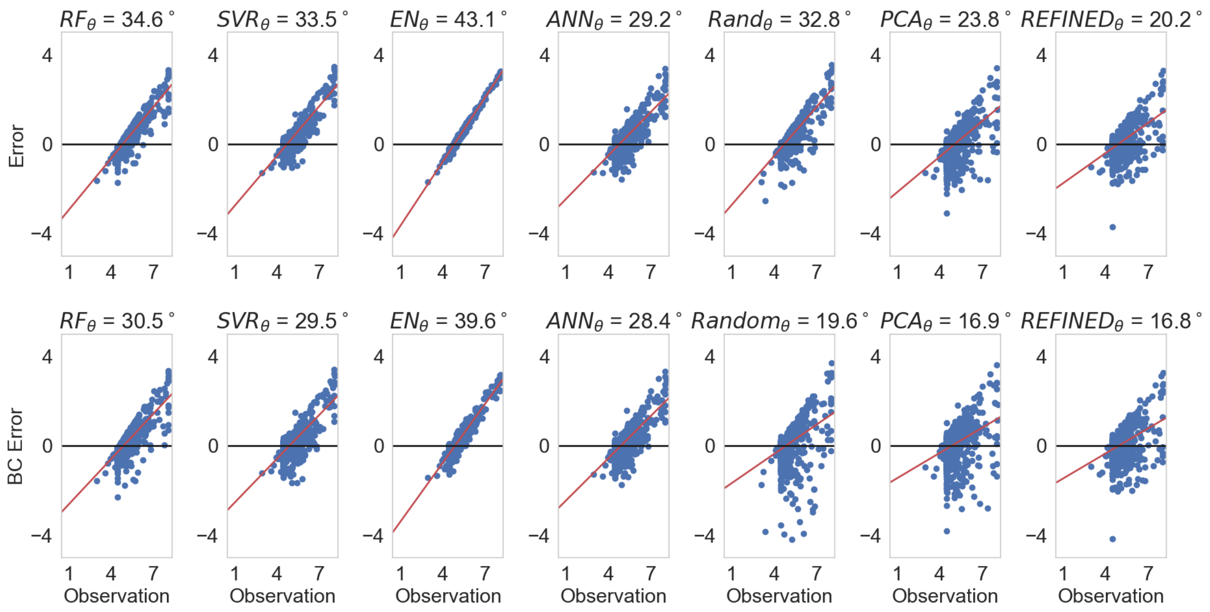

Supplementary Figure 11: Residual plots for seven different models using *SNB_78* cell line data to observe the effects of Bias Correction (BC). First row: Before BC, $\text{NRMSE}_{\text{RF}} = 0.842$, $\text{NRMSE}_{\text{SVR}} = 0.847$, $\text{NRMSE}_{\text{EN}} = 0.964$, $\text{NRMSE}_{\text{ANN}} = 0.822$, $\text{NRMSE}_{\text{Random-CNN}} = 0.864$, $\text{NRMSE}_{\text{PCA-CNN}} = 0.917$, $\text{NRMSE}_{\text{REFINED-CNN}} = 0.784$. Second row: After BC, $\text{NRMSE}_{\text{RF}} = 0.830$, $\text{NRMSE}_{\text{SVR}} = 0.836$, $\text{NRMSE}_{\text{EN}} = 0.937$, $\text{NRMSE}_{\text{ANN}} = 0.817$, $\text{NRMSE}_{\text{Random-CNN}} = 0.832$, $\text{NRMSE}_{\text{PCA-CNN}} = 0.840$, $\text{NRMSE}_{\text{REFINED-CNN}} = 0.763$.

Supplementary Table 3: Comparing REFINED-CNN classifier with six competing classifiers using *McNemar's test* to asses statistical significance (*p*-value) of the prediction across selected cell lines

Cell lines	LR	RF	SVM	ANN	Random-CNN	PCA-CNN
786_01	0	0	0	0	0	0
A4981	0	0	0	0	0	0
A549_ATCC1	0	0	0	0	0	0
ACHN1	0	0	0	0	0	0
BT_5491	5.15E-231	7.01E-192	1.59E-204	5.66E-219	1.01E-261	2.08E-258
CAKI_11	0	0	0	0	0	0
CCRF_CEM1	9.55E-299	2.45E-237	1.01E-261	0	0	0
COLO_2051	0	0	0	0	0	0
DLD_11	2.21E-221	8.24E-230	1.28E-231	3.37E-226	4.22E-227	1.05E-227
DMS_1141	7.28E-158	9.54E-153	1.77E-161	6.25E-148	5.42E-166	5.55E-163
DMS_2731	5.74E-188	3.59E-189	3.42E-195	3.01E-182	7.89E-177	1.92E-180
DU_1451	1.42E-306	1.09E-252	2.15E-283	0	0	0
EKVX1	0	0	0	0	0	0
HCC_29981	0	0	0	0	0	0
MDA_MB_4351	2.21E-221	7.18E-189	1.67E-198	1.22E-237	1.95E-295	4.20E-286
NCI_ADR_RES1	2.23E-249	1.52E-210	6.91E-223	1.36E-253	8.20E-289	2.10E-286
SNB_781	1.64E-229	1.57E-235	1.91E-239	5.03E-234	3.37E-226	1.00E-233

(a)

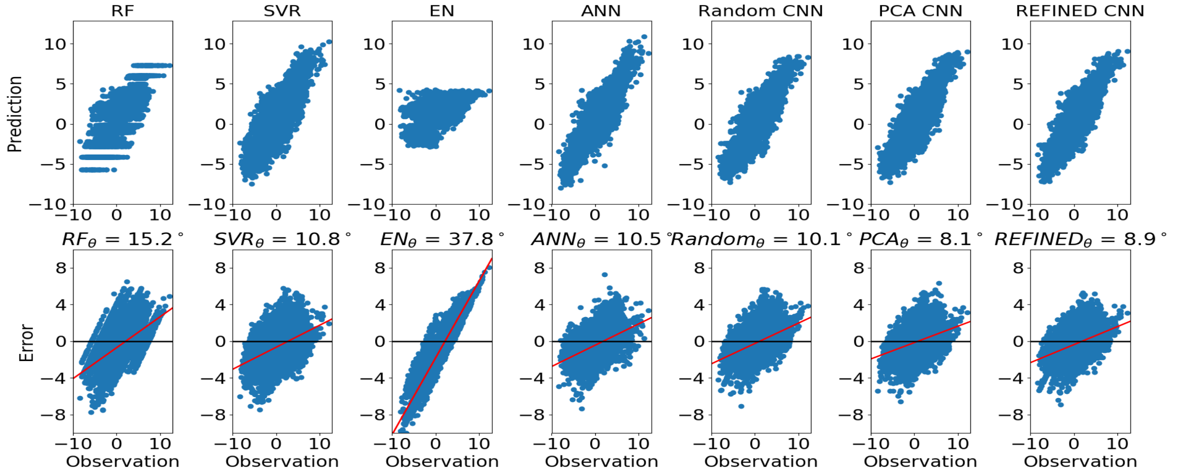

(b)

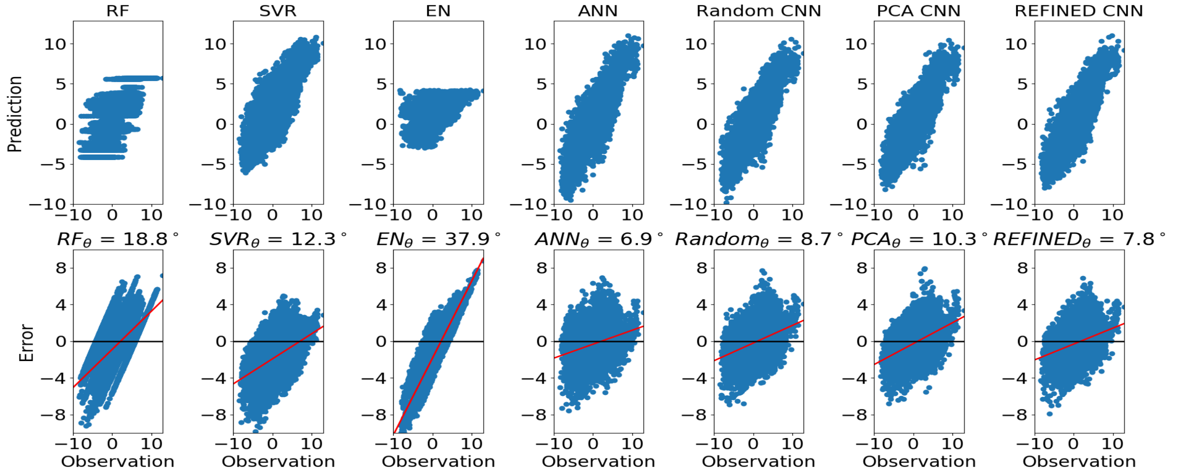

(c)

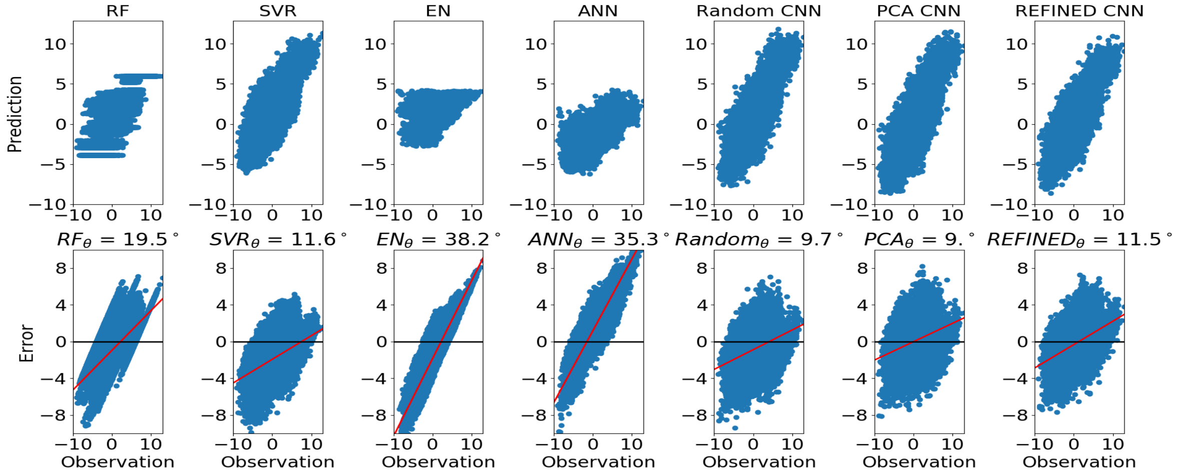

Supplementary Figure 12: Prediction vs. observation and residual vs. observation scatter plots for seven different models, where each model is trained with a percentage of available data. *a.* Training sample size = 80%. *b.* Training sample size = 50%. *c.* Training sample size = 20%.

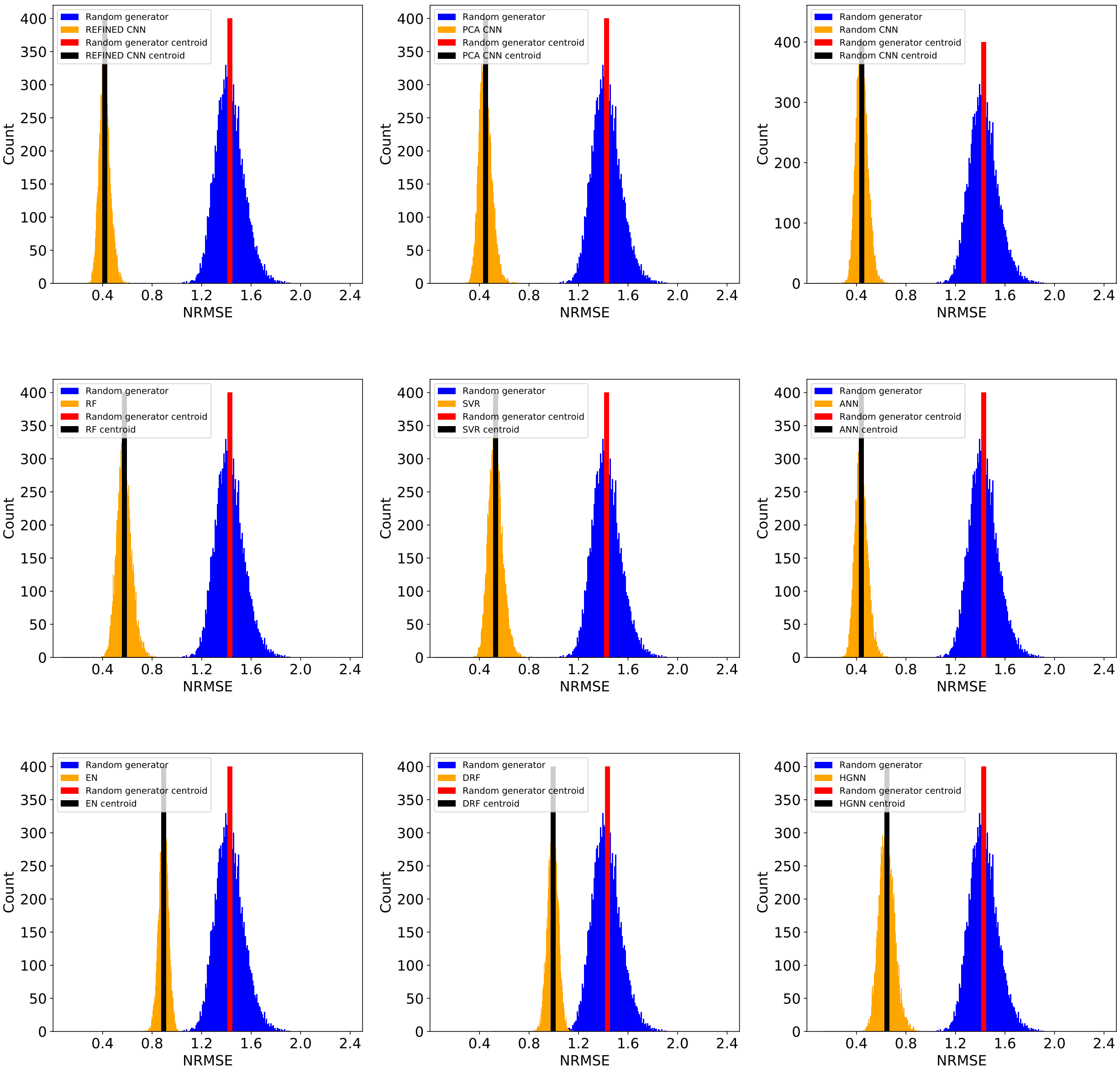

Supplementary Figure 13: Distribution of normalized root mean square error (NRMSE) values of all nine models drawn from the Gap statistics test for GDSC cell lines with the associated cluster centroids as vertical bars.

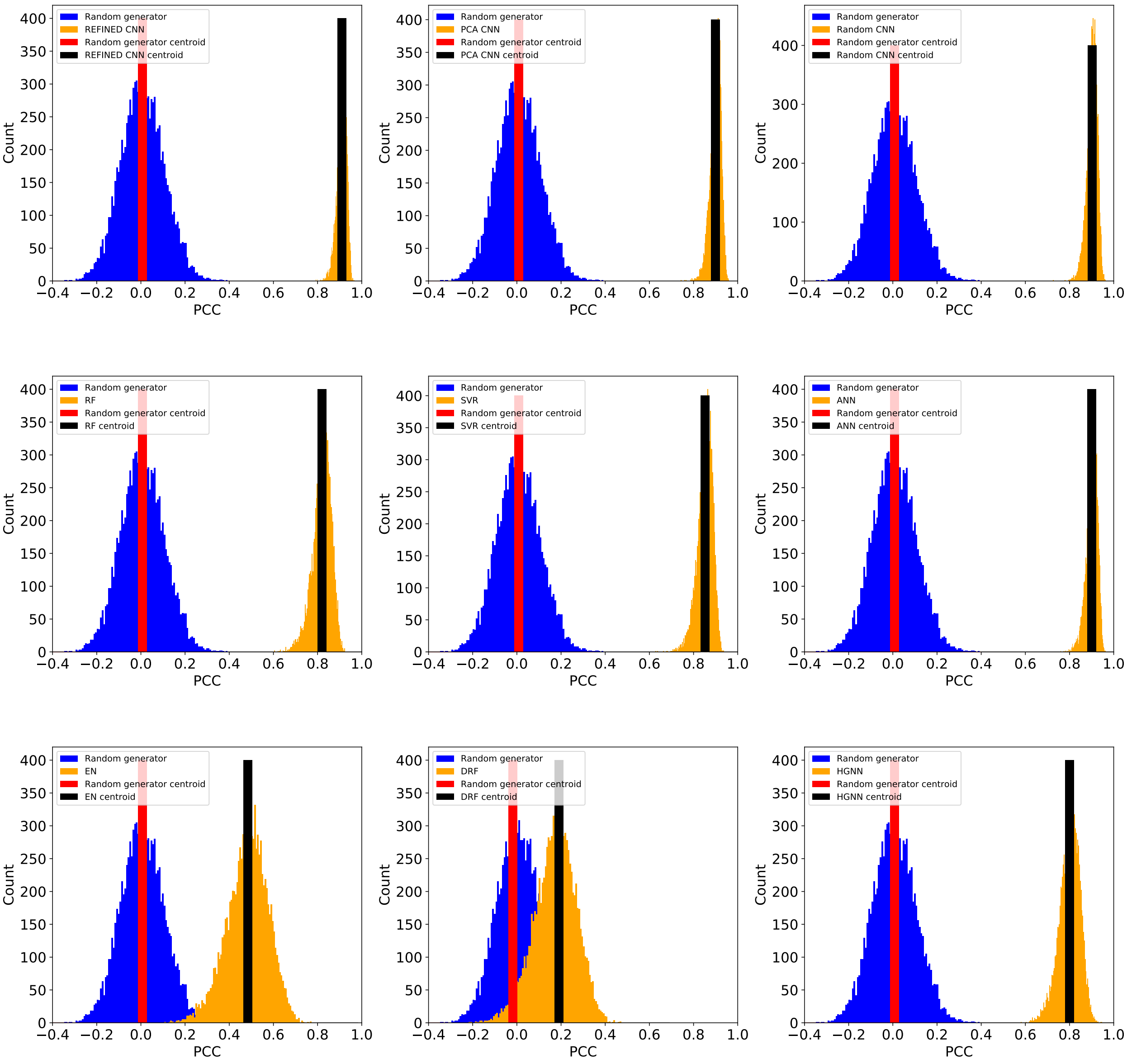

Supplementary Figure 14: Distribution of Pearson correlation coefficient (PCC) values of all nine models drawn from the Gap statistics test for GDSC cell lines with the associated cluster centroids as vertical bars.

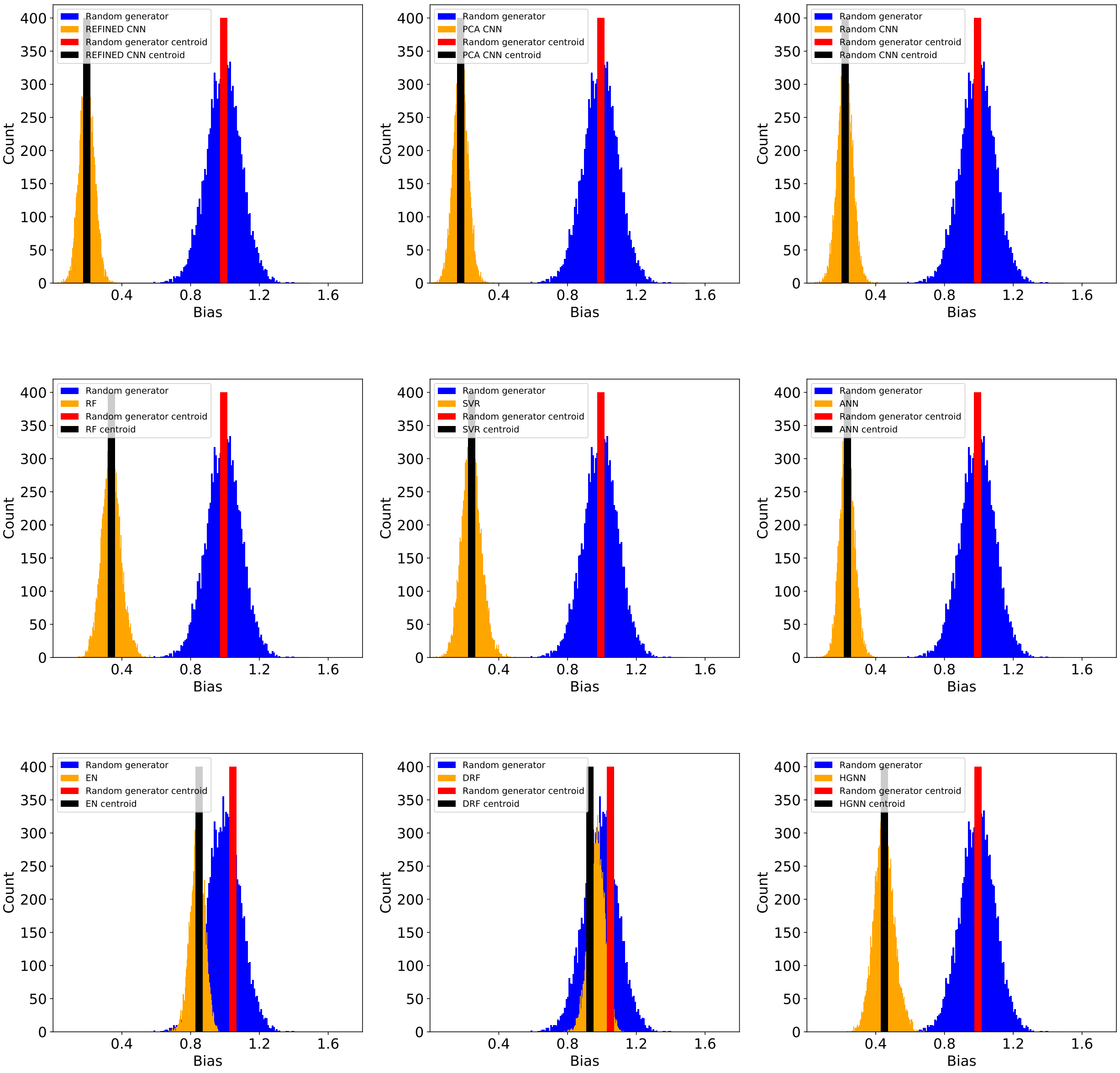

Supplementary Figure 15: Distribution of Bias values of all nine models drawn from the Gap statistics test for GDSC cell lines with the associated cluster centroids as vertical bars.

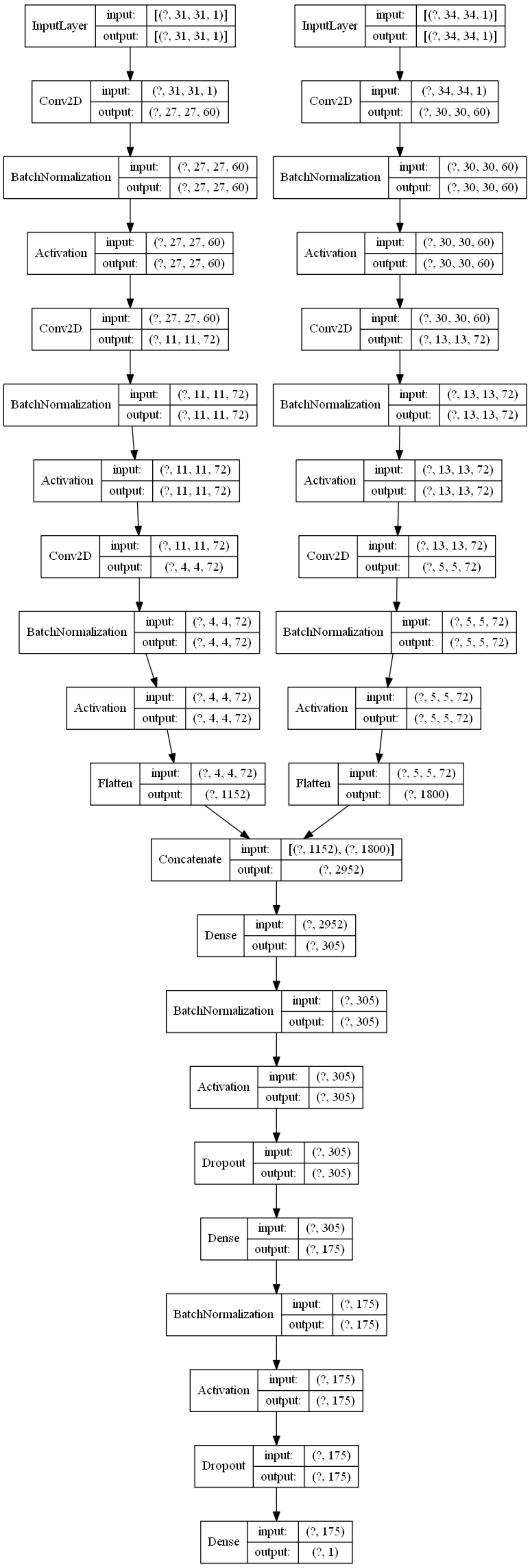

Supplementary Figure 16: CNN architecture used for training the GDSC dataset. Plot created by *graphviz* utility of Keras [3].

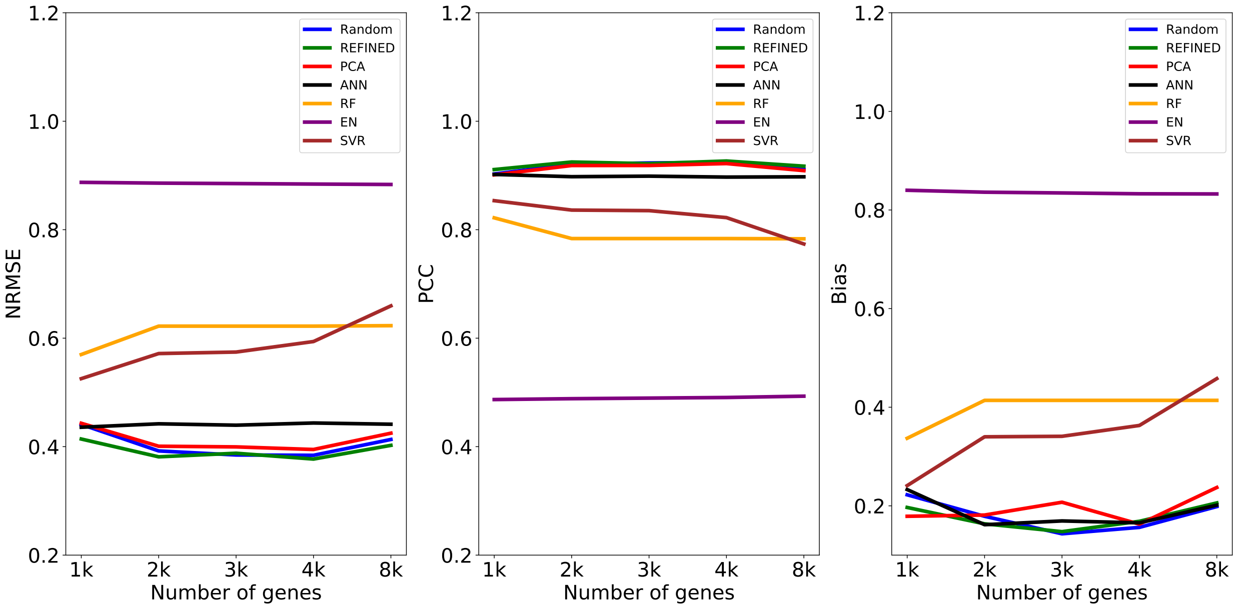

Supplementary Figure 17: Effect of feature size (*i.e.*, number of genes) in GDSC modeling.

Supplementary Table 4: Comparison of sensitivity prediction with seven regression models*

Cell lines	Random-CNN			PCA-CNN			REFINED-CNN			RF			SVR			ANN			EN		
	NRMSE	PCC	Bias	NRMSE	PCC	Bias	NRMSE	PCC	Bias	NRMSE	PCC	Bias	NRMSE	PCC	Bias	NRMSE	PCC	Bias	NRMSE	PCC	Bias
CCRF_CEM	0.868	0.536	0.818	0.884	0.529	0.671	**0.774**	**0.653**	**0.493**	0.893	0.465	0.839	0.874	0.521	0.750	0.828	0.563	0.705	0.978	0.259	0.973
COLO_205	0.958	0.538	0.709	0.823	0.601	0.564	**0.741**	**0.686**	**0.448**	0.892	0.467	0.837	0.867	0.535	0.746	0.811	0.587	0.680	0.974	0.288	0.968
DU_145	0.839	0.572	0.594	0.882	0.555	0.553	**0.786**	**0.647**	**0.458**	0.903	0.434	0.838	0.882	0.507	0.773	0.842	0.539	0.713	0.976	0.268	0.970
EKVX	0.867	0.553	0.579	0.894	0.572	0.514	**0.804**	**0.618**	**0.535**	0.904	0.433	0.842	0.881	0.503	0.769	0.848	0.530	0.713	0.978	0.252	0.972
HCC_2998	0.930	0.578	0.744	0.961	0.535	0.628	**0.774**	**0.654**	**0.447**	0.880	0.488	0.815	0.858	0.542	0.740	0.820	0.572	0.662	0.968	0.312	0.961
MDA_MB_435	0.884	0.532	0.760	0.982	0.557	0.518	**0.787**	**0.651**	**0.469**	0.912	0.412	0.849	0.897	0.477	0.781	0.858	0.514	0.732	0.982	0.234	0.977
SNB_78	0.864	0.516	0.730	0.917	0.544	0.528	**0.784**	**0.652**	**0.448**	0.842	0.558	0.769	0.847	0.555	0.745	0.822	0.573	0.648	0.964	0.352	0.958
NCI_ADR_RES	0.945	0.470	0.751	0.834	0.580	0.565	**0.798**	**0.638**	**0.475**	0.908	0.434	0.860	0.903	0.476	0.799	0.857	0.518	0.737	0.984	0.275	0.981
786_0	0.877	0.558	0.604	0.944	0.599	0.543	**0.752**	**0.665**	**0.450**	0.887	0.481	0.832	0.878	0.521	0.767	0.832	0.571	0.707	0.974	0.289	0.968
A498	0.845	0.604	0.712	0.883	0.576	0.545	**0.785**	**0.635**	**0.433**	0.890	0.465	0.827	0.859	0.551	0.750	0.840	0.567	0.700	0.972	0.312	0.967
A549_ATCC	0.913	0.548	0.560	0.814	0.590	0.623	**0.769**	**0.645**	**0.536**	0.870	0.511	0.806	0.857	0.555	0.743	0.810	0.592	0.692	0.969	0.308	0.962
ACHN	0.830	0.571	0.616	0.837	0.562	0.732	**0.747**	**0.665**	**0.560**	0.880	0.494	0.822	0.868	0.529	0.759	0.816	0.585	0.675	0.975	0.284	0.970
BT_549	0.941	0.529	0.595	0.885	0.545	0.769	**0.843**	**0.584**	**0.560**	0.888	0.478	0.832	0.870	0.514	0.755	0.840	0.545	0.714	0.977	0.279	0.972
CAKI_1	0.866	0.561	0.589	0.886	0.475	0.753	**0.775**	**0.640**	**0.423**	0.901	0.444	0.845	0.885	0.507	0.773	0.863	0.537	0.692	0.982	0.241	0.977
DLD_1	0.923	0.620	0.611	0.812	0.584	0.666	**0.781**	**0.625**	**0.499**	0.847	0.557	0.783	0.867	0.529	0.763	0.824	0.568	0.667	0.975	0.270	0.968
DMS_114	0.873	0.546	0.575	0.953	0.565	0.487	**0.738**	**0.680**	**0.484**	0.832	0.568	0.746	0.834	0.571	0.689	0.804	0.596	0.631	0.985	0.332	0.958
DMS_273	0.810	0.587	0.652	0.909	0.586	0.619	**0.758**	**0.670**	**0.454**	0.829	0.568	0.735	0.860	0.538	0.728	0.817	0.578	0.645	0.984	0.318	0.955
Mean	0.884	0.554	0.659	0.888	0.562	0.605	**0.776**	**0.647**	**0.481**	0.880	0.486	0.816	0.870	0.525	0.755	0.831	0.561	0.689	0.976	0.287	0.968

*Bold values indicate the best performances.

Supplementary Table 5: Comparison of sensitivity prediction with five different Random-CNN regression models for the NCI60 dataset where each model is represented by the associated random seed for input generation and mean denotes the mean prediction for all five models

Cell lines	NRMSE						PCC						Bias					
	Seed 5	Seed 4	Seed 3	Seed 2	Seed 1	Mean	Seed 5	Seed 4	Seed 3	Seed 2	Seed 1	Mean	Seed 5	Seed 4	Seed 3	Seed 2	Seed 1	Mean
MDA_MB_435	0.990	0.873	0.957	0.850	0.860	0.906	0.470	0.600	0.529	0.609	0.580	0.558	0.836	0.495	0.815	0.712	0.579	0.687
SNB_78	0.827	0.830	0.741	0.774	0.815	0.798	0.574	0.560	0.690	0.648	0.610	0.616	0.623	0.667	0.437	0.510	0.511	0.550
NCI_ADR_RES	0.866	0.859	0.843	1.140	0.979	0.938	0.500	0.519	0.565	0.542	0.503	0.526	0.760	0.757	0.645	0.500	0.578	0.648
786_0	0.850	0.854	1.057	0.820	0.879	0.892	0.576	0.609	0.551	0.609	0.583	0.586	0.662	0.702	0.508	0.698	0.732	0.660
A498	0.847	0.828	0.853	0.792	0.848	0.834	0.534	0.589	0.545	0.613	0.594	0.575	0.708	0.611	0.728	0.607	0.706	0.672
A549_ATCC	0.786	0.812	0.775	0.809	0.847	0.806	0.622	0.584	0.635	0.607	0.625	0.615	0.569	0.680	0.635	0.547	0.471	0.580
ACHN	0.835	0.836	0.859	0.825	0.960	0.863	0.575	0.604	0.556	0.567	0.585	0.577	0.592	0.520	0.740	0.683	0.503	0.608
BT_549	0.831	0.938	0.867	0.867	0.931	0.887	0.560	0.444	0.570	0.545	0.467	0.517	0.696	0.794	0.691	0.603	0.704	0.698
CAKI_1	0.825	0.824	0.956	0.814	0.880	0.860	0.578	0.569	0.496	0.585	0.527	0.551	0.674	0.709	0.785	0.639	0.778	0.717
CCRF_CEM	0.844	0.844	1.535	0.940	0.832	0.999	0.544	0.592	0.505	0.521	0.559	0.544	0.687	0.569	0.461	0.621	0.656	0.599
COLO_205	0.891	0.830	0.804	0.859	0.851	0.847	0.582	0.601	0.602	0.572	0.594	0.590	0.540	0.672	0.655	0.583	0.690	0.628
DLD_1	1.142	0.861	0.775	0.796	0.781	0.871	0.481	0.576	0.644	0.626	0.629	0.591	0.648	0.562	0.514	0.555	0.641	0.584
DMS_114	0.802	0.899	0.855	0.821	0.763	0.828	0.620	0.544	0.631	0.589	0.648	0.607	0.705	0.666	0.538	0.684	0.581	0.635
DMS_273	0.786	0.766	0.793	0.782	0.783	0.782	0.620	0.666	0.662	0.658	0.639	0.649	0.644	0.540	0.505	0.556	0.622	0.573
DU_145	0.994	0.886	1.258	0.883	0.797	0.964	0.544	0.548	0.513	0.505	0.628	0.548	0.560	0.615	0.520	0.713	0.526	0.587
EKVX	0.821	0.851	0.891	1.078	0.813	0.891	0.576	0.560	0.539	0.560	0.598	0.567	0.624	0.681	0.681	0.486	0.604	0.616
HCC_2998	0.816	0.814	0.859	0.818	0.793	0.820	0.595	0.612	0.566	0.582	0.613	0.594	0.654	0.603	0.611	0.670	0.618	0.631
Mean	0.868	0.847	0.922	0.863	0.848	0.870	0.562	0.575	0.576	0.585	0.587	0.577	0.658	0.638	0.616	0.610	0.618	0.628

(a)

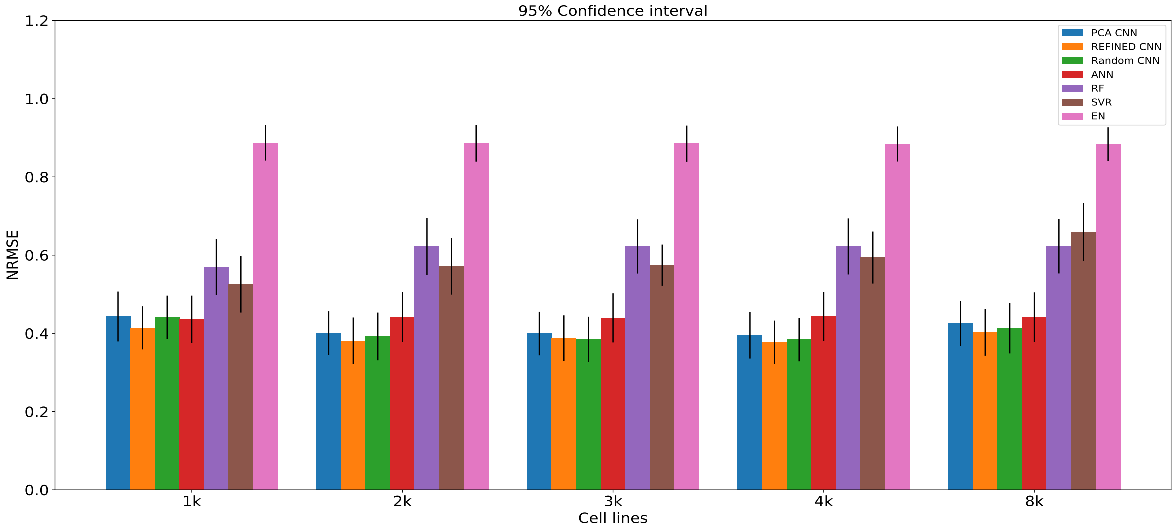

(b)

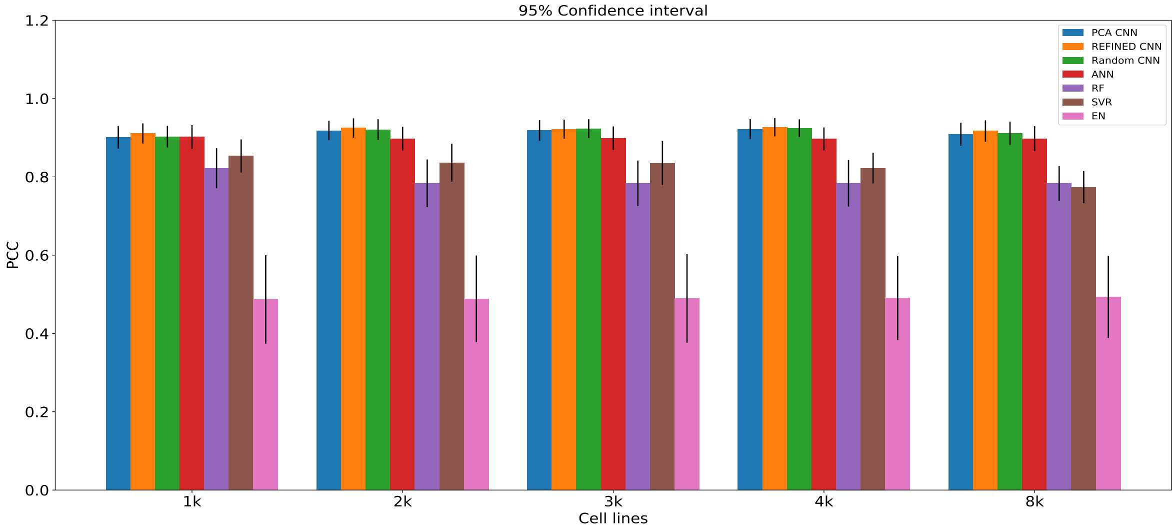

(c)

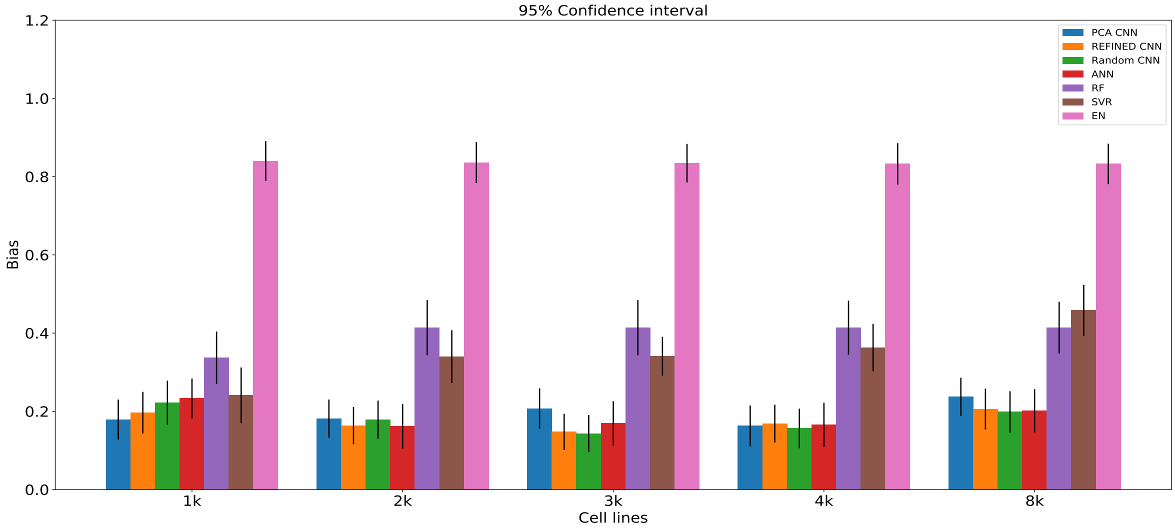

Supplementary Figure 18: 95% **confidence intervals (95% CI) of the evaluation metrics for each model trained on GDSC cell lines.** *a.* 95% CI for normalized rootmean square error (NRMSE). *b.* 95% CI for Pearson correlation coefficient (PCC). *c.* 95% CI for Bias. The gene expression feature size associated with each barchart group is defined under the group PCA-CNN (blue), REFINED-CNN (khaki), Random-CNN (green), ANN (red), RF (purple), SVR (brown) and EN (pink).

(a)

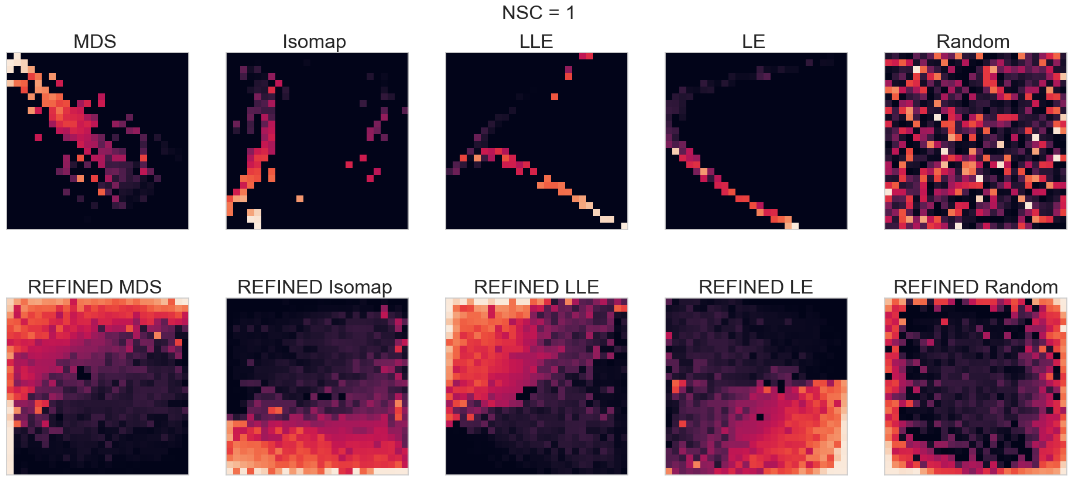

(b)

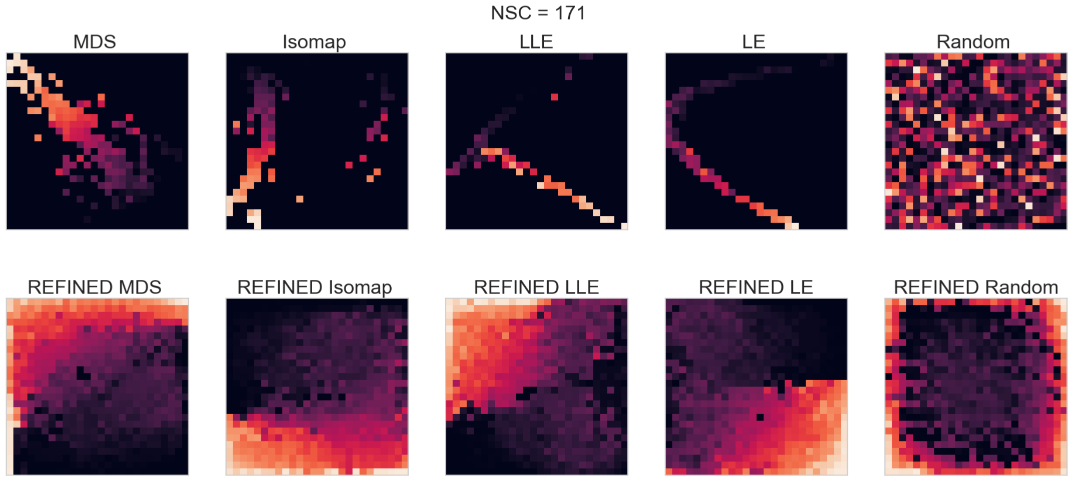

(c)

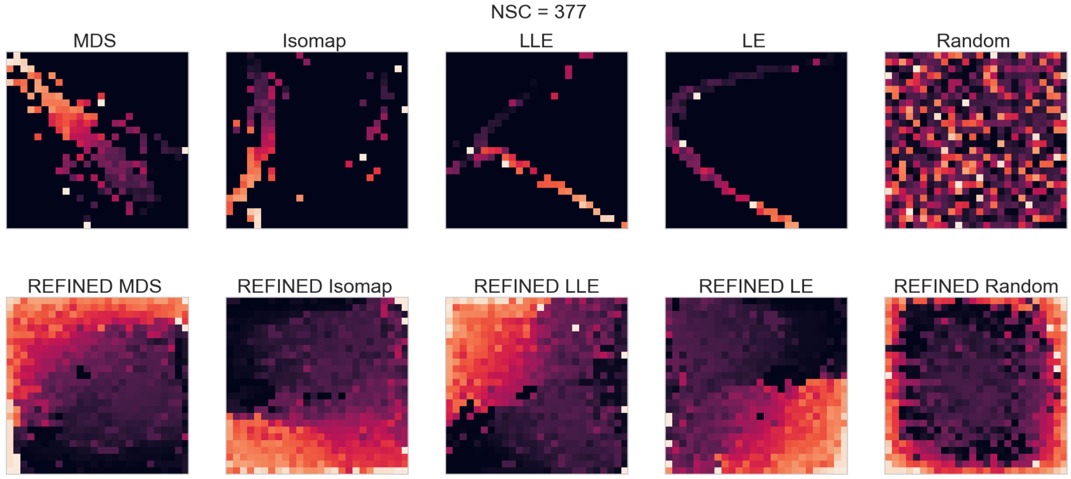

Supplementary Figure 19: Comparison of 2D feature representations by various dimensionality reduction (DR) techniques with REFINED images initialized with corresponding DR representation image. *a.* NSC = 1. *b.* NSC = 171. *c.* NSC = 377. In each subfigure, first row: Images created using different DR techniques, and second row: Images created using REFINED initialized with different DR techniques. Here, the different NSC values correspond to different drugs

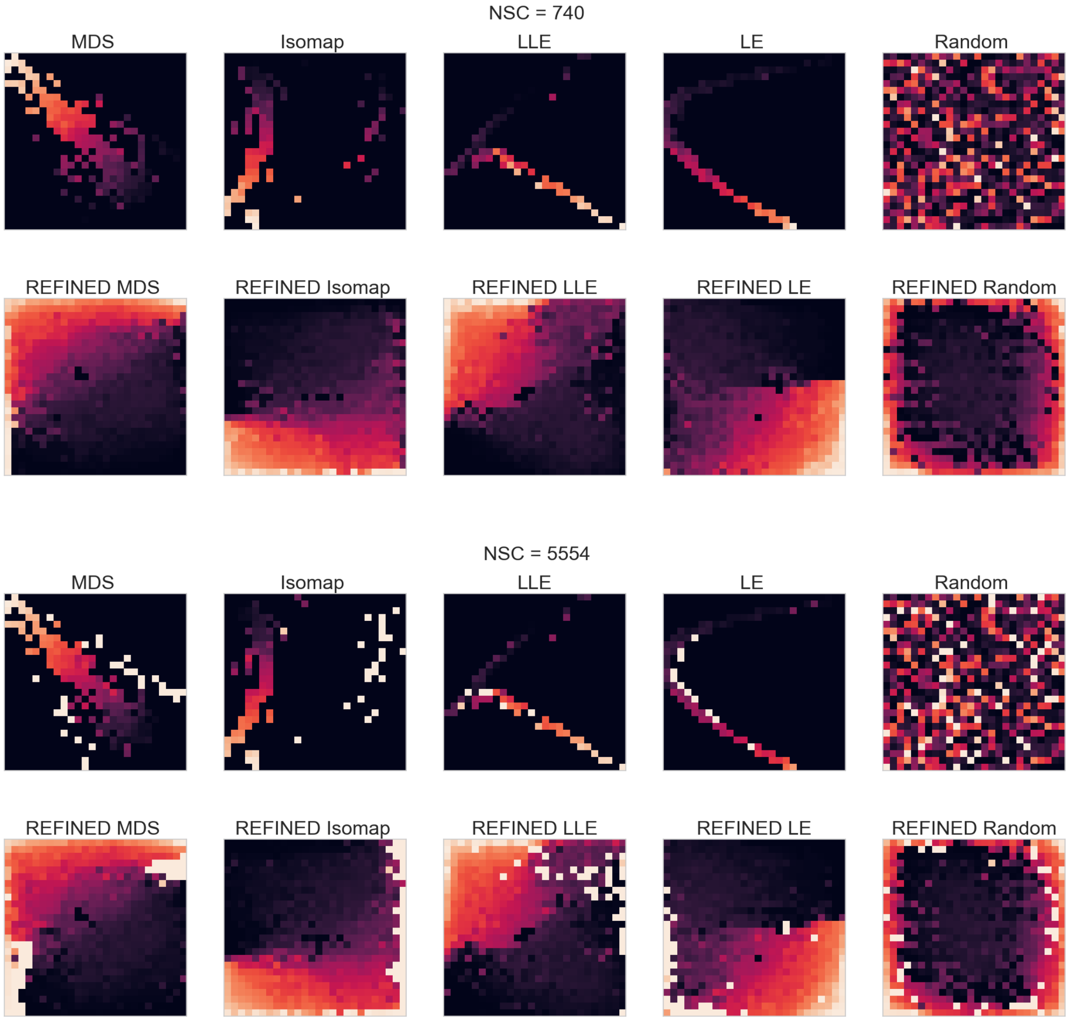

Supplementary Figure 20: Comparison of 2D feature representations by various dimensionality reduction (DR) techniques with REFINED images initialized with corresponding DR representation image. *a.* NSC = 740. *b.* NSC = 5554. In each subfigure, first row: Images created using different DR techniques, and second row: Images created using REFINED initialized with different DR techniques. Here, the different NSC values correspond to different drugs

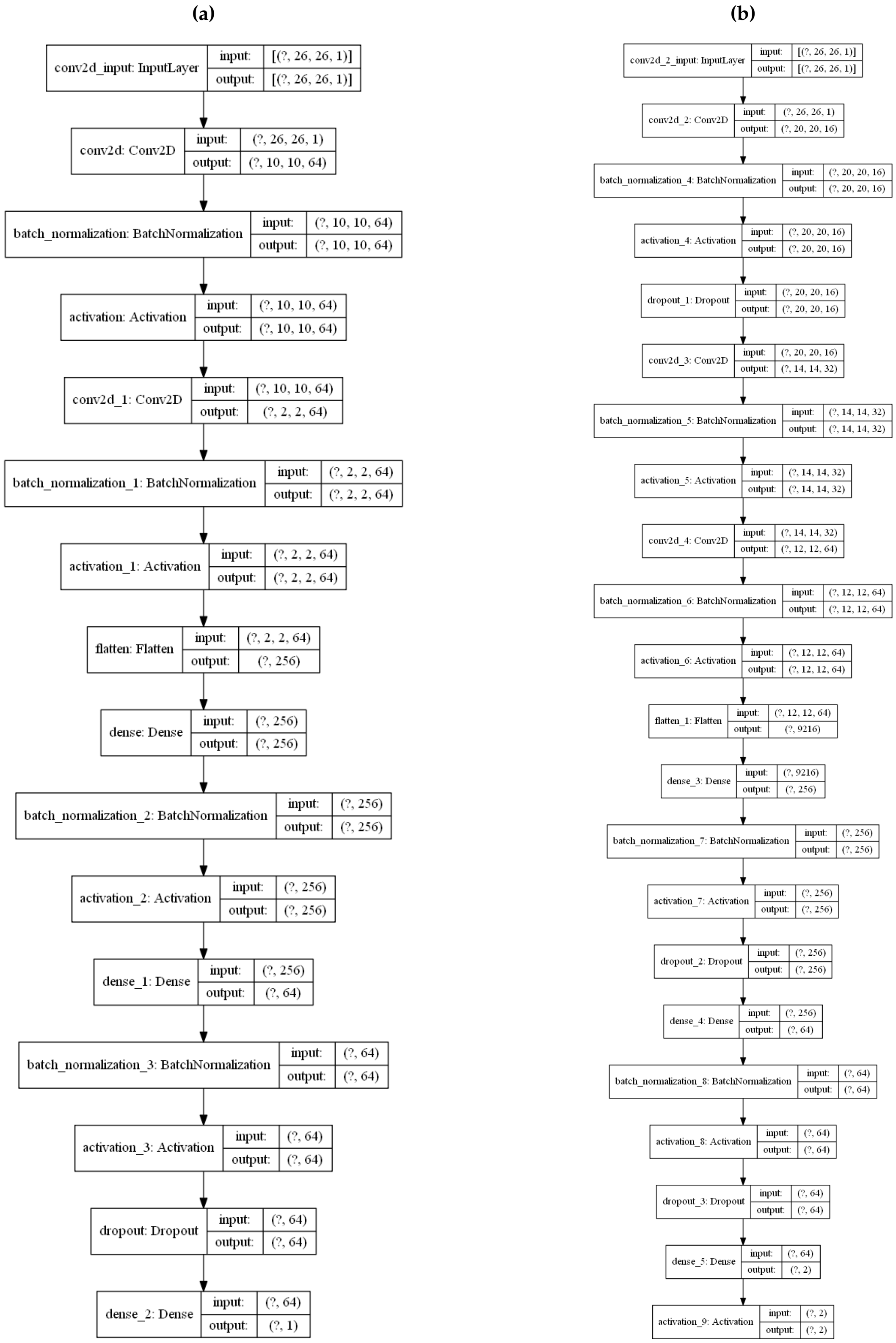

Supplementary Figure 21: CNN architectures used for training the NCI60 dataset. *a.* Network architecture for regression tasks. *b.* Network architecture for classification tasks. Plot created by *graphviz* utility of Keras [3].

(a)

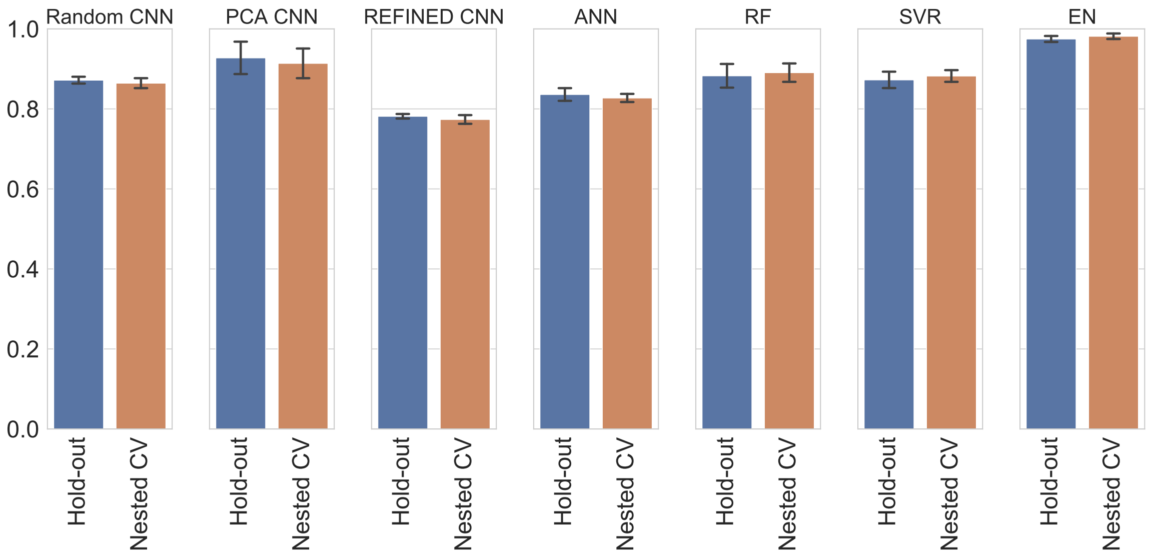

(b)

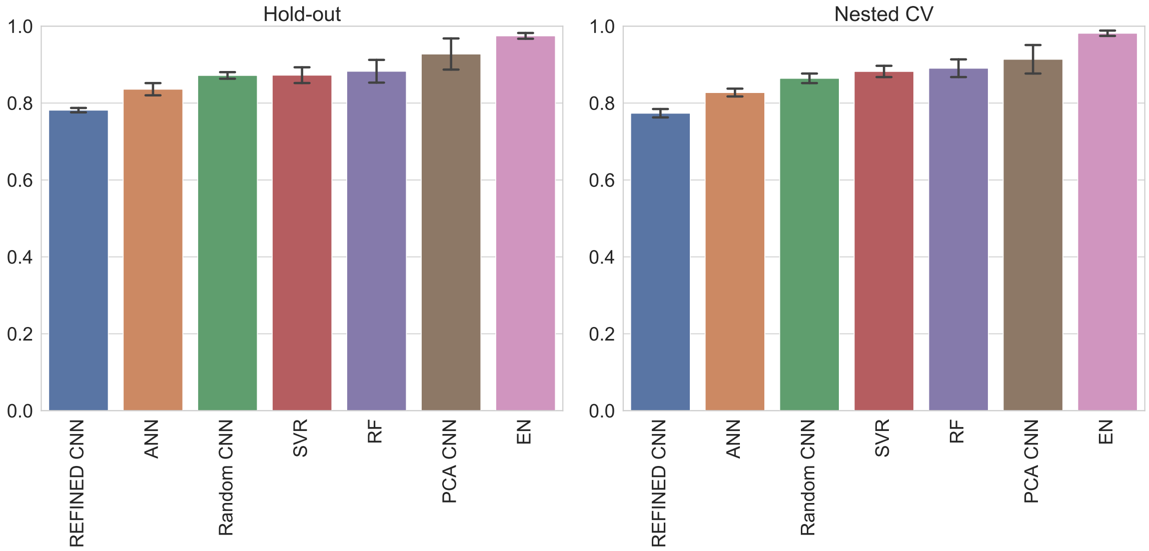

Supplementary Figure 22: Hold-out vs. nested cross-validation (NCV) error estimation performances. *a.* The pairwise comparison of the models shows minimal difference between the two error estimation approaches (Hold-out (blue) and Nested CV (khaki)). *b.* The trend comparison also indicates that the comparative performances of the models do not change whether Hold-out or NCV is used. The colorbar associated with each model is denoted under the bar with colors (REFINED-CNN (blue), ANN (khaki), Random-CNN (green), SVR (red), RF (purple), PCA-CNN (brown) and EN (pink)). The randomly selected NCI60 cell lines for this comparison are CCRF_CEM, MDA_MB_435, and SNB_78 with $\sim 47,000$, $\sim 37,000$ and $\sim 14,000$ samples (*i.e.*, drugs applied to the cell line, see Table 11), respectively

Supplementary Table 6: Comparison of REFINED-CNN performance with six competing models *via* the analyses of different statistical measures *a* Robustness analysis where each cell represents the amount (%) for which REFINED-CNN outperforms the paired model and *b* Gap statistics analysis where larger Gap values indicate better performances*

(a)

Cell lines	REFIEND-CNN vs. PCA-CNN			REFIEND-CNN vs. Random-CNN			REFIEND-CNN vs. ANN			REFIEND-CNN vs. SVR			REFIEND-CNN vs. RF			REFIEND-CNN vs. EN		
	NRMSE %	PCC %	Bias %	NRMSE %	PCC %	Bias %	NRMSE %	PCC %	Bias %	NRMSE %	PCC %	Bias %	NRMSE %	PCC %	Bias %	NRMSE %	PCC %	Bias %
786_0	100	94.3	81.5	99.9	99.7	95.2	99.3	98.7	100	100	100	100	100	100	100	100	100	100
A498	99.8	91.8	95.4	100	100	100	96.8	96.1	99.8	99.5	98.1	100	100	100	100	100	100	100
A549_ATCC	88.8	90.3	98.4	100	98.1	72.9	87.7	88.6	100	99.6	98.3	100	99.8	99.9	100	100	100	100
ACHN	99.8	99.0	100	99.2	98.9	93.7	98.8	97.9	99.7	100	99.8	100	100	100	100	100	100	100
BT_549	88.3	80.6	100	98.9	91.9	81.8	45.0	83.6	100	80.2	95.2	100	91.2	99.2	100	100	100	100
CAKI_1	99.8	100	100	100	95.2	100	99.5	99.5	100	100	99.9	100	100	100	100	100	100	100
DLD_1	78.1	78.1	88.4	100	98.8	100	91.5	90.8	99.4	99.9	98.7	100	100	100	100	100	100	100
DMS_114	100	99.3	51.8	100	100	100	98.8	99.1	100	100	100	100	100	100	100	100	100	100
DMS_273	100	97.6	100	99.9	87.4	82.0	94.3	99.2	100	99.6	99.8	100	98.9	98.9	100	100	100	100
HCC_2998	100	99.7	68.7	99.5	98.9	95.3	79.2	89.7	82.4	96.1	96.9	99.4	96.8	97.2	100	100	100	100
CCRF_CEM	100	99.9	100	84	94.0	100	88.5	95.5	100	99.8	99.6	100	94.8	97.1	100	100	100	100
COLO_205	95.1	93.7	99.8	99.4	99.6	100	99.8	100	100	100	100	100	100	100	100	100	100	100
DU_145	98.8	99	98.8	100	100	100	96.4	99.9	100	99.8	100	100	99.8	100	100	100	100	100
EKVX	96.9	85	91.6	100	80.1	100	67.3	86.7	100	93.1	96.2	100	98.6	99.8	100	100	100	100
MDA_MB_435	100	99.9	87.7	99.8	100	100	98.9	100	100	99.9	100	100	100	100	100	100	100	100
NCI_ADR_RES	98	98.9	98.7	100	100	100	97.1	100	100	100	100	100	100	100	100	100	100	100
SNB_78	99.3	98	88.5	96.7	99.4	100	82.7	94.5	100	92	97	100	91.3	96.8	100	100	100	100
Mean	96.63	94.42	91.14	98.66	96.59	95.35	89.51	95.28	98.90	97.62	98.79	99.96	98.31	99.35	100.00	100.00	100.00	100.00

(b)

Cell lines	REFINED-CNN Gap			PCA-CNN Gap			Random-CNN Gap			RF Gap			SVR Gap			ANN Gap			EN Gap		
	NRMSE	PCC	Bias	NRMSE	PCC	Bias	NRMSE	PCC	Bias	NRMSE	PCC	Bias	NRMSE	PCC	Bias	NRMSE	PCC	Bias	NRMSE	PCC	Bias
786_0	**0.735**	**0.661**	0.468	0.544	0.593	**0.565**	0.604	0.554	0.407	0.598	0.481	0.407	0.611	0.520	0.241	0.652	0.566	0.301	0.478	0.095	0.145
A498	**0.699**	**0.630**	0.400	0.597	0.570	0.468	0.501	0.469	**0.588**	0.592	0.462	**0.588**	0.628	0.549	0.260	0.640	0.562	0.309	0.477	0.327	0.039
A549_ATCC	**0.716**	**0.641**	**0.479**	0.668	0.585	0.389	0.564	0.541	0.453	0.615	0.507	0.453	0.631	0.552	0.265	0.676	0.588	0.319	0.477	0.179	0.037
ACHN	**0.730**	**0.658**	**0.450**	0.641	0.556	0.274	0.647	0.567	0.396	0.596	0.490	0.396	0.612	0.525	0.250	0.661	0.580	0.334	0.471	0.222	0.036
BT_549	**0.806**	**0.581**	**0.454**	0.616	0.548	0.235	0.555	0.529	0.422	0.613	0.477	0.422	0.633	0.514	0.253	0.660	0.541	0.294	0.495	0.283	0.029
CAKI_1	**0.708**	**0.635**	0.483	0.597	0.476	0.254	0.595	0.571	**0.591**	0.583	0.445	**0.591**	0.602	0.506	0.234	0.618	0.534	0.315	0.477	0.222	0.140
DLD_1	**0.710**	**0.622**	**0.403**	0.676	0.580	0.348	0.609	0.522	0.190	0.594	0.462	0.190	0.617	0.519	0.256	0.663	0.558	0.301	0.476	0.320	0.143
DMS_114	**0.764**	**0.673**	0.530	0.555	0.562	**0.535**	0.633	0.513	0.300	0.608	0.464	0.300	0.638	0.533	0.263	0.693	0.583	0.327	0.492	0.288	0.168
DMS_273	**0.725**	**0.667**	**0.561**	0.563	0.581	0.397	0.557	0.610	0.513	0.631	0.555	0.513	0.618	0.530	0.251	0.656	0.565	0.348	0.470	0.331	0.029
HCC_2998	**0.713**	**0.648**	0.417	0.521	0.529	0.401	0.607	0.540	**0.441**	0.654	0.566	**0.441**	0.655	0.569	0.326	0.681	0.591	0.384	0.481	0.077	0.029
RF_CEM	**0.711**	**0.649**	**0.521**	0.601	0.529	0.341	0.672	0.582	0.361	0.653	0.568	0.361	0.628	0.544	0.285	0.666	0.576	0.370	0.479	0.244	0.134
OLO_205	**0.727**	**0.681**	**0.562**	0.661	0.621	0.446	0.625	0.567	0.417	0.564	0.437	0.417	0.588	0.507	0.233	0.625	0.536	0.294	0.461	0.243	0.132
DU_145	**0.708**	**0.643**	**0.556**	0.612	0.551	0.462	0.605	0.470	0.153	0.591	0.435	0.153	0.618	0.499	0.240	0.647	0.526	0.296	0.490	0.318	0.150
EKVX	**0.684**	**0.613**	0.475	0.591	0.567	**0.501**	0.557	0.579	0.258	0.606	0.481	0.258	0.634	0.538	0.266	0.668	0.567	0.344	0.481	0.296	0.140
MDA_MB_435	**0.699**	**0.647**	**0.545**	0.403	0.509	0.496	0.604	0.531	0.245	0.572	0.409	0.245	0.593	0.476	0.228	0.627	0.511	0.274	0.481	0.329	0.138
NCI_ADR_RES	**0.701**	**0.633**	**0.537**	0.621	0.541	0.448	0.554	0.472	0.259	0.593	0.434	0.259	0.602	0.476	0.210	0.644	0.516	0.269	0.494	0.428	0.147
SNB_78	**0.716**	**0.649**	**0.575**	0.568	0.539	0.496	0.633	0.526	0.297	0.653	0.554	0.297	0.656	0.556	0.276	0.672	0.568	0.371	0.479	0.282	0.183
Mean	**0.721**	**0.643**	**0.495**	0.590	0.555	0.415	0.596	0.538	0.370	0.607	0.484	0.370	0.622	0.524	0.255	0.656	0.557	0.321	0.480	0.264	0.107

*Bold values indicate the best performances.

Supplementary Table 7: REFINED-CNN performance for different training sample size (given as percentages of the actual size) for five randomly selected NCI60 cell lines

Training size	CCRF_CEM		EKVX		MDA_MB_435		NCI_ADR_RES		SNB_78	
	NRMSE	PCC	NRMSE	PCC	NRMSE	PCC	NRMSE	PCC	NRMSE	PCC
20%	1.279	0.373	0.899	0.498	0.949	0.454	0.953	0.464	0.876	0.537
40%	0.985	0.478	0.881	0.511	0.911	0.494	0.958	0.536	0.825	0.590
60%	0.903	0.538	0.849	0.525	0.839	0.575	0.857	0.528	0.806	0.591
80%	0.774	0.653	0.804	0.618	0.787	0.651	0.798	0.638	0.784	0.652

Supplementary Table 8: Comparison of REFINED-CNN performance with six competing models *via* the analyses of different statistical measures *a* Robustness analysis where each cell represents the amount (%) for which REFINED-CNN outperforms the paired model and *b* Gap statistics analysis where larger Gap values indicate better performances

(a)

Comparison	NRMSE	PCC	Bias
REFINED-CNN vs. ANN	62.23	60.68	73.49
REFINED-CNN vs. RF	98.39	98.19	97.95
REFINED-CNN vs. SVR	93.83	93.36	73.47
REFINED-CNN vs. EN	100	100	100
REFINED-CNN vs. PCA-CNN	66.58	62.04	68.85
REFINED-CNN vs. Random-CNN	65.78	69.77	65.27
REFINED-CNN vs. DRF	100	100	100
REFINED-CNN vs. HGNN	99.87	98.81	99.99

(b)

Model	NRMSE	PCC	Bias
REFINED-CNN	1.010	0.902	0.853
PCA-CNN	0.980	0.892	0.871
Random-CNN	0.983	0.895	0.828
RF	0.852	0.812	0.710
SVR	0.898	0.844	0.808
ANN	0.988	0.893	0.815
EN	0.535	0.476	0.200
DRF	0.432	0.184	0.120
HGNN	0.782	0.792	0.550

Supplementary Table 9: Effect of feature size (number of genes) in GDSC modeling

Metrics	#Genes = 1000			#Genes = 2000			#Genes = 3000			#Genes = 4000			#Genes = 8000		
	NRMSE	PCC	Bias	NRMSE	PCC	Bias	NRMSE	PCC	Bias	NRMSE	PCC	Bias	NRMSE	PCC	Bias
EN	0.887	0.487	0.840	0.886	0.488	0.836	0.885	0.490	0.835	0.884	0.491	0.833	0.883	0.493	0.833
RF	0.570	0.822	0.337	0.622	0.784	0.414	0.622	0.784	0.414	0.622	0.784	0.414	0.623	0.783	0.414
SVR	0.525	0.854	0.241	0.572	0.836	0.340	0.574	0.835	0.341	0.594	0.822	0.363	0.660	0.774	0.458
ANN	0.436	0.902	0.233	0.442	0.898	0.162	0.440	0.899	0.169	0.444	0.897	0.166	0.441	0.898	0.201
Random-CNN	0.441	0.903	0.222	0.392	0.921	0.179	0.385	0.923	0.143	0.384	0.924	0.156	0.413	0.911	0.198
PCA-CNN	0.443	0.901	0.179	0.401	0.918	0.181	0.400	0.918	0.207	0.395	0.922	0.163	0.425	0.909	0.237
REFINED-CNN	0.414	0.911	0.197	0.381	0.925	0.163	0.388	0.922	0.148	0.377	0.927	0.169	0.402	0.917	0.206

Supplementary Table 10: Comparison of CNN performance using images created by different dimensionality reduction (DR) techniques from the Ablation study and REFINED images initialized with these DR techniques *a* Without search optimization and *b* Using hill climbing

(a)

Cell line	Isomap			LLE			MDS			LE			Random		
	NRMSE	PCC	Bias	NRMSE	PCC	Bias	NRMSE	PCC	Bias	NRMSE	PCC	Bias	NRMSE	PCC	Bias
CCRF_CEM	0.962	0.470	0.701	0.980	0.233	0.963	0.831	0.622	0.667	0.797	0.614	0.561	0.868	0.536	0.818
COLO_205	0.891	0.584	0.514	1.262	0.000	1.000	0.787	0.622	0.59	0.785	0.641	0.483	0.958	0.538	0.709
DU_145	0.868	0.507	0.724	0.979	0.406	0.835	0.864	0.592	0.523	0.806	0.614	0.533	0.839	0.572	0.594
EKVX	0.853	0.572	0.665	1.021	0.397	0.812	0.867	0.545	0.587	0.841	0.570	0.574	0.867	0.553	0.579
HCC_2998	0.830	0.565	0.630	0.832	0.591	0.562	0.825	0.628	0.467	0.929	0.550	0.545	0.93	0.578	0.744
MDA_MB_435	0.911	0.453	0.773	0.922	0.499	0.643	1.019	0.429	0.748	0.821	0.586	0.632	0.884	0.532	0.76
SNB_78	0.828	0.576	0.598	0.859	0.519	0.754	0.819	0.588	0.684	0.817	0.585	0.684	0.864	0.516	0.73
NCI_ADR_RES	1.315	0.470	0.481	0.895	0.474	0.793	0.938	0.488	0.746	0.824	0.576	0.616	0.945	0.47	0.751
786_0	0.820	0.602	0.546	0.827	0.574	0.677	0.814	0.647	0.66	0.852	0.618	0.477	0.877	0.558	0.604
A498	0.892	0.573	0.534	1.018	0.045	0.998	0.844	0.582	0.681	0.797	0.618	0.537	0.845	0.604	0.712
A549_ATCC	0.801	0.614	0.557	0.851	0.559	0.724	0.896	0.599	0.765	0.825	0.631	0.470	0.913	0.548	0.56
ACHN	1.109	0.494	0.602	0.914	0.462	0.815	0.789	0.631	0.514	0.900	0.601	0.449	0.83	0.571	0.616
BT_549	0.850	0.530	0.701	0.890	0.465	0.768	0.926	0.499	0.764	0.835	0.562	0.624	0.941	0.529	0.595
CAKI_1	0.874	0.548	0.649	1.439	0.376	0.781	0.935	0.546	0.546	0.799	0.609	0.569	0.866	0.561	0.589
DLD_1	0.880	0.587	0.531	0.902	0.468	0.829	0.786	0.629	0.533	0.792	0.636	0.652	0.923	0.62	0.611
DMS_114	0.830	0.577	0.599	0.854	0.539	0.709	0.847	0.619	0.467	0.898	0.602	0.503	0.873	0.546	0.575
DMS_273	0.780	0.639	0.529	0.813	0.598	0.588	0.801	0.62	0.52	0.771	0.639	0.579	0.81	0.587	0.652
Mean	0.900	0.551	0.608	0.956	0.424	0.780	0.858	0.582	0.616	0.829	0.603	0.558	0.884	0.554	0.659

(b)

Cell line	REFINED_Isomap			REFINED_LLE			REFINED_MDS			REFINED_LE			REFINED_Random		
	NRMSE	PCC	Bias	NRMSE	PCC	Bias	NRMSE	PCC	Bias	NRMSE	PCC	Bias	NRMSE	PCC	Bias
CCRF_CEM	0.799	0.630	0.498	0.781	0.638	0.515	0.774	0.653	0.493	0.790	0.633	0.510	0.816	0.586	0.725
COLO_205	0.752	0.673	0.493	0.765	0.652	0.509	0.741	0.686	0.448	0.782	0.658	0.433	0.812	0.606	0.662
DU_145	0.858	0.628	0.443	0.825	0.613	0.531	0.786	0.647	0.458	0.822	0.621	0.595	0.935	0.518	0.604
EKVX	0.790	0.634	0.504	0.839	0.587	0.526	0.804	0.618	0.535	0.824	0.601	0.622	0.998	0.528	0.580
HCC_2998	0.758	0.668	0.456	0.834	0.623	0.465	0.774	0.654	0.447	0.772	0.660	0.451	0.833	0.608	0.454
MDA_MB_435	0.817	0.622	0.495	0.893	0.574	0.688	0.787	0.651	0.469	0.824	0.609	0.559	0.811	0.627	0.586
SNB_78	0.827	0.636	0.436	0.831	0.626	0.458	0.784	0.652	0.448	0.755	0.661	0.547	0.846	0.542	0.778
NCI_ADR_RES	0.791	0.632	0.517	0.823	0.603	0.583	0.798	0.638	0.475	0.811	0.608	0.547	0.810	0.542	0.593
786_0	0.751	0.678	0.504	0.763	0.663	0.467	0.752	0.665	0.450	0.756	0.672	0.451	0.784	0.637	0.661
A498	0.799	0.624	0.508	0.785	0.662	0.435	0.785	0.635	0.433	0.789	0.633	0.569	0.787	0.631	0.669
A549_ATCC	0.754	0.673	0.472	0.779	0.650	0.465	0.769	0.645	0.536	0.750	0.675	0.469	0.858	0.556	0.473
ACHN	0.768	0.684	0.526	0.766	0.656	0.503	0.747	0.665	0.560	0.756	0.659	0.530	0.922	0.585	0.551
BT_549	0.812	0.594	0.583	0.821	0.606	0.521	0.843	0.584	0.560	0.801	0.609	0.585	0.822	0.591	0.708
CAKI_1	0.785	0.639	0.491	0.817	0.590	0.608	0.775	0.640	0.423	0.816	0.617	0.510	0.836	0.571	0.666
DLD_1	0.729	0.687	0.506	0.821	0.622	0.527	0.781	0.625	0.499	0.750	0.680	0.433	0.832	0.594	0.537
DMS_114	0.764	0.671	0.510	0.736	0.690	0.431	0.738	0.680	0.484	0.749	0.676	0.467	0.857	0.563	0.576
DMS_273	0.754	0.667	0.492	0.752	0.693	0.478	0.758	0.670	0.454	0.722	0.701	0.453	0.866	0.546	0.463
Mean	0.783	0.649	0.496	0.802	0.632	0.512	0.776	0.647	0.481	0.781	0.645	0.514	0.849	0.578	0.605

REFINED_Isomap: REFINED approach with Isomap mapping as initialization instead of Bayesian MDS, REFINED_LLE: REFINED approach with LLE mapping as initialization instead of Bayesian MDS, REFINED_MDS: proposed REFINED approach with Bayesian MDS mapping as initialization, REFINED_LE: REFINED approach with LE mapping as initialization instead of Bayesian MDS, REFINED_Random: REFINED approach with random mapping as initialization instead of Bayesian MDS.

Supplementary Table 11: Number of drugs applied to each cell line in NCI60

Cell line	#Drugs (Samples)
CCRF_CEM	47,571
COLO_205	49,844
DU_145	36,622
EKVX	47,732
HCC_2998	45,192
MDA_MB_435	36,868
SNB_78	14,006
NCI_ADR_RES	37,156
786_0	49,453
A498	43,853
A549_ATCC	50,703
ACHN	49,760
BT_549	33,466
CAKI_1	47,410
DLD_1	14,650
DMS_114	14,937
DMS_273	13,728
**Total**	632,951

Supplementary References

[1] Shoemaker, R. H. The nci60 human tumour cell line anticancer drug screen. *Nat. Rev. Cancer* **6,** 813-823 (2006).

[2] Yang, W. et al. Genomics of drug sensitivity in cancer (gdsc): a resource for therapeutic biomarker discovery in cancer cells. *Nuc. Acids Res.* **41,** 955-961 (2012).

[3] Chollet, F. et al. Keras. `https://keras.io/` (2015).
